# Mitogen-Activated Protein Kinase Cascades in Plant Hormone Signaling

**DOI:** 10.3389/fpls.2018.01387

**Published:** 2018-10-08

**Authors:** Przemysław Jagodzik, Małgorzata Tajdel-Zielinska, Agata Ciesla, Małgorzata Marczak, Agnieszka Ludwikow

**Affiliations:** ^1^Department of Plant Physiology, Institute of Experimental Biology, Faculty of Biology, Adam Mickiewicz University in Poznań, Poznań, Poland; ^2^Department of Biotechnology, Institute of Molecular Biology and Biotechnology, Faculty of Biology, Adam Mickiewicz University in Poznań, Poznań, Poland

**Keywords:** MAP kinase cascade, auxin, abscisic acid, jasmonic acid, salicilic acid, ethylene, brassinosteroids, gibberellin

## Abstract

Mitogen-activated protein kinase (MAPK) modules play key roles in the transduction of environmental and developmental signals through phosphorylation of downstream signaling targets, including other kinases, enzymes, cytoskeletal proteins or transcription factors, in all eukaryotic cells. A typical MAPK cascade consists of at least three sequentially acting serine/threonine kinases, a MAP kinase kinase kinase (MAPKKK), a MAP kinase kinase (MAPKK) and finally, the MAP kinase (MAPK) itself, with each phosphorylating, and hence activating, the next kinase in the cascade. Recent advances in our understanding of hormone signaling pathways have led to the discovery of new regulatory systems. In particular, this research has revealed the emerging role of crosstalk between the protein components of various signaling pathways and the involvement of this crosstalk in multiple cellular processes. Here we provide an overview of current models and mechanisms of hormone signaling with a special emphasis on the role of MAPKs in cell signaling networks.

**One-sentence summary:** In this review we highlight the mechanisms of crosstalk between MAPK cascades and plant hormone signaling pathways and summarize recent findings on MAPK regulation and function in various cellular processes.

## Introduction

Mitogen-activated protein kinases (MAPKs) are one of the largest group of transferases, catalyzing phosphorylation of appropriate protein substrates on serine or threonine residues. MAPK cascades are among the most common mechanisms by which cell functions are regulated and are evolutionarily conserved throughout the eukaryotes, including plants, fungi and mammals (Zanke et al., 1996; Ligterink and Hirt, 2001; Xu et al., 2017). In plants, they play essential roles in the transduction of environmental and developmental signals. MAPKs are present in the cytoplasm and nucleus, and take part in different cellular processes including growth, development and stress responses (Seguí-Simarro et al., 2005; Pitzschke et al., 2009; Gupta and Chakrabarty, 2013; Sheikh et al., 2013; Danquah et al., 2015; Wang Z. et al., 2015). By regulating MAPK cascades, cells are able to respond to a range of stresses caused by high or low temperature, UV radiation, ozone, reactive oxygen species, drought, high or low osmolarity, heavy metals, wounding and pathogen infections (Sinha et al., 2011; Opdenakker et al., 2012; Danquah et al., 2014; de Zelicourt et al., 2016). Importantly, hormones such as auxin (AUX), abscisic acid (ABA), jasmonic acid (JA), salicylic acid (SA), ethylene (ET), brassinosteroids (BR), and gibberellins (GA) are known to influence signaling through MAPK cascades (Mishra et al., 2006; Rodriguez et al., 2010; Smekalova et al., 2013; Hettenhausen et al., 2014; Lu et al., 2015). In this review we highlight the mechanisms of crosstalk between MAPK cascades and plant hormone signaling pathways and summarize recent findings on MAPK regulation and function in various cellular processes.

## MAPK Cascades in Plants

**FIGURE 1:**
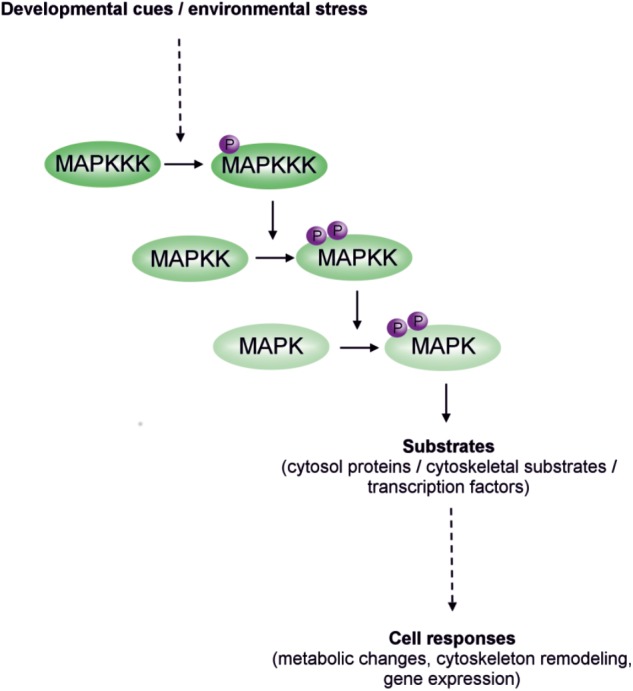
Schematic representation of MAPK cascade.

The transduction and enhancement of input signals by MAPK cascades involves three types of kinase: mitogen activated protein (MAP) kinase kinase kinases (MAPKKKs; also known as MAP3Ks or MEKKs), MAP kinase kinases (MKKs; also known as MAP2Ks or MEKs) and MAP kinases (MAPKs; also known as MPK) (**Figure 1**). MAP kinase kinase kinase kinases (MAPKKKKs) have also been identified in plants (Colcombet and Hirt, 2008; Raja et al., 2017). The first signal transduction step is the activation of a MAPKKKK or MAPKKK by stimulation of plasma membrane receptors. The MAPKKK then activates a downstream MAPKK by phosphorylation of two serine or threonine residues in the S/T-X_5_-S/T (X is any amino acid) motif of its activation loop. Once activated, the MAPKK behaves as a dual-specificity kinase, which phosphorylates a MAPK on the threonine and tyrosine residues in the T-X-Y motif of an activation loop located between subdomains VII and VIII of its catalytic domain (Rodriguez et al., 2010; Hettenhausen et al., 2014). MAPKs are serine/threonine kinases that activate various effector proteins in the cytoplasm or nucleus, including other kinases, enzymes, cytoskeletal proteins or transcription factors (Khokhlatchev et al., 1998; Rodriguez et al., 2010). Interactions between the kinases are mediated by docking sites in the enzymes themselves and/or by external scaffolding proteins. Such a series of phosphorylation events is termed a MAPK cascade.

Nearly 110 genes encoding MAPK cascade kinases have been identified in the *Arabidopsis thaliana* genome; these genes encode 20 MAPK, 10 MAPKK, and 80 MAPKKK proteins (Colcombet and Hirt, 2008; de Zelicourt et al., 2016; Raja et al., 2017). There are comparable numbers of MAPK cascade kinase genes in other plant species, e.g., the rice (*Oryza sativa*) genome contains 17 *MAPK*, 8 *MAPKK* and 75 *MAPKKK* genes (Xiong et al., 2001; Singh et al., 2012; Wankhede et al., 2013), the tomato (*Solanum lycopersicum*) genome 17 *MAPK* (Mohanta et al., 2015), 5 *MAPKK* and 89 *MAPKKK* genes (Wu et al., 2014) and the maize (*Zea mays*) genome 19 *MAPK*, 9 *MAPKK* and 74 *MAPKKK* genes (Kong et al., 2013) (**Supplementary Tables 1**, **2**). Similar gene sets in other plant genomes and the presence of MAPK cascade kinases in a huge range of species indicate that they are evolutionarily conserved among higher plants (Danquah et al., 2014).

The MAPKKK family forms the largest and most heterogeneous group of MAPK cascade components (Danquah et al., 2014). Based on sequence analysis, Arabidopsis MAPKKKs can be divided into three main classes: MEKK-like (21 members), Raf-like (48 members) and ZIK-like (11 members; also known as WNK [with no lysine (K)] MAPKKKs (Danquah et al., 2014).

MEKK-like kinases fall into four subgroups (A1 – A4) (MAPK Group, 2002). Subgroup A1 in *A. thaliana* includes four functionally characterized protein kinases: MAP/ERK kinase kinase 1 (AtMEKK1, also called AtMAPKKK8), AtMEKK2 (AtMAPKKK9), AtMEKK3 (AtMAPKKK10), and AtMEKK4 (AtMAPKKK11) (MAPK Group, 2002). MEKK-like kinases of subgroup A1 have also been identified in other species, e.g., *Nicotiana benthamiana* MAPKKKβ (NbMAPKKKβ) and *Brassica napus* MAPKKK8 (BnaMAPKKK8, also known as BnMAP3Kβ1) (Jouannic et al., 1999; Hashimoto et al., 2012; Sun et al., 2014). Subgroup A2 consists of AtMAP3Kα (AtMAPKKK3), AtMAP3Kγ (AtMAPKKK5), AtYODA (AtMAPKKK4), BnaMAPKKK3 (also known as BnaMAP3Kα1), NbMAPKKKα and NbMAPKKKγ (Hashimoto et al., 2012; Sun et al., 2014), while subgroup A3 comprises AtANP1 (also called AtMAPKKK1), AtANP2 (AtMAPKKK2), AtANP3 (AtMAPKKK12) and their ortholog *Nicotiana* protein kinase 1 (NtNPK1) (MAPK Group, 2002; Sun et al., 2014). Subgroup A4, as the last functionally characterized subgroup of MEKK-like kinases, consists of AtMAP3Kε1 (AtMAPKKK7), AtMAP3Kε2 (AtMAPKKK6) and AtMAPKKK13–21 in *A. thaliana*, and BnaMAPKKK6 (BnMAP3Kε1) and BnaMAPKKK19-20 in *Brassica napus* (MAPK Group, 2002; Chaiwongsar et al., 2012; Sun et al., 2014).

Raf-like MAPKKKs have been classified into groups B and C (MAPK Group, 2002; Wu et al., 2014). Two of the best-characterized Raf-like kinases, Arabidopsis constitutive triple response 1 (AtCTR1, also known as AtRaf1) and enhanced disease resistance 1 (AtEDR1, also known as AtRaf2), together with orthologs such as *O. sativa* OsEDR1 (OsMAPKKK1) and drought-hypersensitive mutant 1 (OsDSM1) (OsMAPKKK6), and BnaCTR1 and BnaEDR1 (BnaRaf2), are members of group B (MAPK Group, 2002; Shen et al., 2011; Yin et al., 2013; Danquah et al., 2014; Sun et al., 2014; Virk et al., 2015). Members of group C remain mostly uncharacterized.

Although both Raf-like and ZIK-like kinases are clearly members of the MAPKKK family, they have not been confirmed to phosphorylate MAPKKs in plants (Danquah et al., 2014; Chardin et al., 2017). However, Sun et al. (2014) have recently shown that Raf-like and ZIK-like kinases interact with MAPKKs in canola (*Brassica napus* L.).

Plant MAPKKs, with the exception of MKK10 homologs, feature a S/T-X_5_-S/T motif in the activation loop (Jonak et al., 2002; MAPK Group, 2002; Doczi et al., 2007, 2012; Danquah et al., 2014; Poyraz, 2015). Some MKK10 homologs, such as ZmMKK10-2 and OsMKK10-2, show only a partial MAPKK consensus motif (R-X_5_-S/T), while others, such as AtMKK10, ZmMKK10-1, OsMKK10-1 and OsMKK10-3, do not have this consensus sequence at all (**Supplementary Figure 1**). Some MAPKKs in green algae, such as *Chlamydomonas reinhardtii* MKK6 (CreinMKK6) and *Volvox carteri* MKK3 (VcMKK3), also lack the consensus motif (**Supplementary Figure 1**).

The Arabidopsis genome contains ten *MAPKK* genes, which have been divided into four groups, A – D (**Supplementary Figure 2** and **Supplementary Table 1**). All AtMAPKKs in group A have also been shown to interact with AtMPK6 and AtMPK11 (**Supplementary Figure 3**) (Meszaros et al., 2006; Lee J.S. et al., 2008). In addition, AtMKK1 activates AtMPK3 and AtMPK12 (Meszaros et al., 2006; Lee et al., 2009), AtMKK2 interacts with AtMPK5, AtMPK10 and AtMPK13 (Teige et al., 2004; Gao et al., 2008; Lee J.S. et al., 2008), while AtMKK6 besides AtMPK4 activates AtMPK13 and interacts with AtMPK12 in yeast cells (Melikant et al., 2004; Lee J.S. et al., 2008; Takahashi et al., 2010; Zeng et al., 2011).

Group A MAPKKs have also been reported in rice, alfalfa (*Medicago sativa*), tobacco (*Nicotiana tabacum* L., *Nicotiana benthamiana*), tomato (*Solanum lycopersicum* L., *Lycopersicon esculentum*,), green algae (*Volvox carteri* and *Chlamydomonas reinhardtii*), lycophyte (*Selaginella moellendorffii*), maize (*Zea mays* L.) and canola (*Brassica napus* L.) (Cardinale et al., 2002; Xie et al., 2012; Liang et al., 2013; Cai et al., 2014; Li X. et al., 2014) (**Supplementary Figure 2** and **Supplementary Table 1**). Group B MAPKKs include AtMKK3 in *A. thaliana* and their homologs in *O. sativa* (Wankhede et al., 2013), *Z. mays* (Liang et al., 2013; Kong et al., 2013), *Brassica napus*, *S. lycopersicum*, *N. tabacum*, *S. moellendorffii*, *C. reinhardtii*, and *V. carteri* (Liang et al., 2013) (**Supplementary Figure 2**). Group C MAPKKs include AtMKK4 and AtMKK5 as well as MKK4 and/or MKK5 proteins in other species (**Supplementary Figure 2**) (Kong et al., 2011; Furuya et al., 2014).

The last category of MAPKKs is group D, which includes the remaining Arabidopsis MAPKKs, such as AtMKK7, AtMKK8, AtMKK9, and AtMKK10 (**Supplementary Figure 2**). AtMKK7 interacts with AtMPK2 (Lee J.S. et al., 2008), AtMPK12 (Lee et al., 2009) and AtMPK15 (Lee J.S. et al., 2008), while AtMKK9 interacts with AtMPK6, AtMPK10 (Lee J.S. et al., 2008), AtMPK12 (Lee et al., 2009), AtMPK17 and AtMPK20 (Lee J.S. et al., 2008). In other species, only homologs of AtMKK8 have not been identified (Kong et al., 2013; Wankhede et al., 2013). Interestingly, AtMKK10, OsMKK10-2 and ZmMKK10-1, family members that lack (partially or completely) the MAPKK consensus motif (**Supplementary Figure 1**), nevertheless interact with MAPKs (Lee J.S. et al., 2008; Singh et al., 2012; Wankhede et al., 2013; Kong et al., 2013) (**Supplementary Figure 3**).

The MAPKs themselves form the last category of MAPK cascade component. MAPKs feature the conserved T-X-Y motif, which is phosphorylated by MAPKKs during signal transduction. Based on sequence similarities, the 20 Arabidopsis MAPKs have been divided into two subtypes, TEY (12 MAPKs) and TDY (8 MAPKs). The MAPKs of the TEY subtype carry a T-E-Y (Thr-Glu-Tyr) motif and can be divided into three groups (A – C). The more evolutionarily distant group D is formed by MAPKs of the TDY subtype, which contain a T-D-Y (Thr-Asp-Tyr) motif at the phosphorylation site (Bigeard and Hirt, 2018) (**Supplementary Table 2**). Recent phylogenetic analysis of MAPKs from 40 plant species revealed that group A, as well as MAPKs carrying the T-E-Y motif, also contains MAPKs sharing a T-Q-Y (Thr-Gln-Tyr) motif, while group B also includes MAPKs carrying M-E-Y (Met-Glu-Tyr), T-E-C (Thr-Glu-Cys) and T-V-Y (Thr-Val-Tyr) motifs (Mohanta et al., 2015) (**Supplementary Figure 4**). Three MAPKs of group C also contain a motif that is different to the typical T-E-Y sequence. These are OlMPK7 from *Ostreococcus lucimarinus*, which carries a T-S-Y (Thr-Ser-Tyr) motif, and *Picea abies* MPK7-1 (PaMPK7-1) and PaMPK20, which both contain a M-S-Y (Met-Ser-Tyr) motif sequence (**Supplementary Figure 4**). A MAPK motif was not found in the sequence of OsMPK20-2 from group D (**Supplementary Figure 4**). Phylogenetic analysis of MAPKs from 40 plant species allowed two additional groups to be distinguished, i.e., E and F, which mainly contain MAPKs of lower eukaryotic and gymnosperm plants (Mohanta et al., 2015). However, our own phylogenetic analysis of MAPKs clearly identified group E MAPKs with T-E-Y, T-D-Y, T-R-M (Thr-Arg-Met), T-E-M (Thr-Ser-Met) and T-Q-M (Thr-Gln-Met) motifs (**Supplementary Figure 4**), but MAPKs such as CrenMPK4-1, *Micromonas pusila* MPK4 (MpMPK4), OlMPK6 and VcMPK4-1 assigned to group F by Mohanta et al. (2015) are actually members of group C (**Supplementary Figure 5**). Therefore, we suggest retaining the group E category, but MAPKs of group F should be incorporated into group C.

Group A TEY MAPKs include AtMPK3, AtMPK6 and AtMPK10 in Arabidopsis, and their homologs in canola, alfalfa, cucumber, tobacco, rice, spruce (*Picea abies*), tomato, and maize (MAPK Group, 2002; Liang et al., 2013; Wankhede et al., 2013; Mohanta et al., 2015; Wang J. et al., 2015) (**Supplementary Table 2**). Group B includes AtMPK4, AtMPK5, AtMPK11-13, whereas AtMPK1, AtMPK2, AtMPK7, AtMPK14 are members of group C. Members of group B and C are also present in others plants including gymnosperms and algae (Liang et al., 2013; Wankhede et al., 2013; Mohanta et al., 2015; Wang J. et al., 2015) (**Supplementary Figure 5** and **Supplementary Table 2**). Group D is formed by MAPKs of the TDY subtype, such as AtMPK8-9 and MPK15-20 (MAPK Group, 2002; Liang et al., 2013; Wankhede et al., 2013; Mohanta et al., 2015; Wang J. et al., 2015) (**Supplementary Table 2**). Group E includes 12 MAPKs from species such as *C. reinhardtii, C. subellipsoidea*, *M. pusila*, *N. tabacum*, *S. moellendorffii*, *P. abies* and *V. carteri* (**Supplementary Figures 4**, **5**).

## MAPK Modules Involed in Auxin Signaling

The phytohormone auxin, indole 3-acetic acid (IAA), plays a crucial role in plant growth and development, including embryogenesis (Friml et al., 2003; Blilou et al., 2005; Morris et al., 2005; Leyser, 2017), organogenesis (Benkova et al., 2003; Reinhardt et al., 2003; Heisler et al., 2005; Smekalova et al., 2014; Contreras-Cornejo et al., 2015; Enders et al., 2017; Wójcikowska and Gaj, 2017; Corredoira et al., 2017; Zhao, 2018), tissue patterning, tropism and growth responses to environmental stimuli (Benkova et al., 2003; Leyser, 2003; Reinhardt et al., 2003; Morris et al., 2004; Simonini et al., 2016; Liu et al., 2017; Kamada et al., 2018). The involvement of auxin in this multiplicity of biological processes results from its regulation of cell division, expansion and differentiation (Chen and Baluska, 2013). Auxin synthesis takes place mainly in the shoot, after which it is distributed directionally throughout the plant. Auxin distribution patterns are asymmetric within tissues and they vary dynamically throughout different developmental stages (Friml et al., 2003; Tanaka et al., 2006; Béziat and Kleine-Vehn, 2018). Since Mizoguchi et al. (1994) observed that auxin can activate MAPKs in tobacco cells, several MAPK cascades have been implicated in the regulation of auxin biosynthesis, transport and signal transduction. However, published studies on the connection between auxin and MAPK signaling have given conflicting results, such that in some cases the same MAPK activities apparently mediate different functions.

### MAPK Pathways as Positive and Negative Regulators of Auxin Signal Transduction

The pioneering work of Mizoguchi et al. (1994) suggested a link between auxin and MAPK activity. They reported that *in vitro* phosphorylation of myelin basic protein (MBP) and a recombinant MAPK by extracts of tobacco BY-2 cells increased when cells were subjected to prior treatment with a high concentration of the synthetic auxin. However, using the same system, Tena and Renaudin (1998) showed that auxin at low concentrations does not induce MBP kinase activity in tobacco cell lines. Activation of MAPK was observed only after treatment with very high concentrations of synthetic auxin and was probably a consequence of cytoplasmic acidification caused by its accumulation (Tena and Renaudin, 1998; Mockaitis and Howell, 2000).

In other early studies, Kovtun et al. (1998) showed that *Nicotiana* protein kinase NPK1, a member of the MAPKKK family in tobacco, initiates a MAPK cascade that negatively regulates early IAA-inducible genes. Recombinant NPK1 was transiently expressed in leaf protoplasts to determine its influence on the activity of the soybean GH3 promoter, which is known to be auxin-responsive (Liu et al., 1994; Kovtun et al., 1998). Overexpression of NPK1 specifically blocked the auxin inducibility of the GH3 promoter, while a MAPK-specific phosphatase (MKP1) was able to abolish this effect (Kovtun et al., 1998). Similar results were obtained using orthologs of NPK1, i.e., Arabidopsis ANP1, ANP2, and ANP3. In an experiment with constitutively active ANPs, transiently overexpressed in protoplasts, it was shown that the ANPs selected can also suppress auxin signaling (Kovtun et al., 2000). ANP1 mediates H_2_O_2_-induced activation of the known stress MAPKs, AtMPK3 and AtMPK6, and the end result of this activation cascade is inhibition of auxin-inducible genes (Kovtun et al., 2000; Hirt, 2000). This evidence for a role of NPK1 and its orthologs in auxin signaling is consistent with their involvement in cytokinesis (Jin et al., 2002; Takahashi et al., 2010). MPK12 is another negative regulator of auxin signaling and its kinase activity increases after auxin treatment. *MPK12* RNAi lines were hypersensitive to auxin in a root growth inhibition assay. Furthermore, IBR5, which mediates crosstalk between the auxin and ABA signaling pathways, has been identified as a specific MPK12 phosphatase (Monroe-Augustus et al., 2003; Lee et al., 2009; Raja et al., 2017).

In contrast to the negative role of MAPK in auxin signaling, Zhao et al. (2013) found that MAPKs positively regulate some auxin genes (e.g., *OsYUCCA4*) under conditions of cadmium stress in rice roots, while other genes (e.g., *OsPINc*) are negatively regulated. The complex relationship between MAPKs and auxin signaling was further studied with respect to cadmium and zinc stresses. Zhao et al. (2014a,b) performed a comprehensive expression analysis of 67 key genes in the auxin signaling pathway. Seven genes were positively regulated by MAPK cascades, namely *OsYUCCA3, OsPIN1c, OsPIN10b, OsPID, OsARF20, OsIAA9* and *OsIAA30*. In addition, 14 genes were negatively regulated by MAPKs (*OsYUCCA1, OsYUCCA2, OsPIN5a, OsPIN5b, OsARF7, OsARF8, OsARF12, OsARF15, OsARF16, OsARF21, OsARF22, OsARF25, OsIAA12*, and *OsIAA15*). It should be emphasized that the combined results of Zhao et al., 2014b indicate that MAPKs function at the interface between H_2_O_2_ and auxin signaling under Cd and Zn stress conditions. This evidence suggests a model where MAPKs regulate auxin distribution through H_2_O_2_, while H_2_O_2_ in turn may act downstream of MAPKs but upstream of the auxin signaling pathway. It would be interesting to investigate the precise MAPK-dependent regulatory mechanisms that facilitate auxin/ROS (reactive oxygen species) regulation.

### MAPK Signaling as a Regulator of Polar Auxin Transport

Polar auxin transport (PAT) is an active process whereby auxin is delivered to specific plant tissues (Tanaka et al., 2006; Petrasek and Friml, 2009; Leyser, 2017; Zhao, 2018). Interestingly, PAT is regulated by PIN proteins and reversible protein phosphorylation, mediated by protein kinases and protein phosphatases, and it can, for example, control the activity of auxin transport proteins (Muday and DeLong, 2001; Dai et al., 2006; Ganguly et al., 2014; Dory et al., 2018). Localization of the plasma membrane localized PIN proteins is also controlled by several MAP kinases including MPK4 and the MKK7/MPK6 module (Jia et al., 2016; Dory et al., 2018). The involvement of MKK7 in PAT was shown by analyses of the Arabidopsis *bud1* mutant, which has significantly fewer lateral roots than wild-type (Mou et al., 2002; Dai et al., 2006) and shows disrupted PAT from shoots into roots, as well as a deficiency in auxin signaling (Reed et al., 1998; Xie et al., 2000; Rogg et al., 2001; Dai et al., 2006). Molecular genetic analysis of *bud1* plants by Dai et al. (2006) revealed increased expression of the *AtMKK7* gene, which results in defective auxin transport, while lowering *AtMKK7* mRNA levels using antisense RNA causes an improvement in auxin transport. Together, these data suggest that AtMKK7 is a negative regulator of PAT (Dai et al., 2006; Zhang X. et al., 2008).

Recently, another module, AtMKK2/AtMPK10, has been implicated in the regulation of PAT. The results of Stanko et al. (2014) may indicate that the AtMKK2/AtMPK10 module regulates auxin transport, with consequences for venation complexity and other developmental phenomena. It seems that at least two MAPK pathways connect auxin to development, but the precise regulatory connections have not yet been fully elucidated.

It is worth mentioning that other interesting links between MAPKs and auxins exist. YODA kinase (MAPKKK4) and MPK6 have been shown to be involved in an auxin-dependent regulation of cell division during post-embryogenic root development. Smekalova et al. (2014), showed that both loss-of-function (*yda1*) and gain-of-function (Δ*Nyda1)* plants exhibit pronounced root phenotypes that result from visibly disorientated cell divisions. Both mutants have elevated endogenous auxin (IAA) levels, and this might be related to their phenotypes. Indeed, because the IAA level is particularly upregulated in Δ*Nyda1* plants, it is tempting to hypothesize that the role of YODA in the elongation of the zygote is to promote auxin signaling. It is known that YODA acts upstream of MPK3/6 in stomatal development (Bergmann et al., 2004; Lukowitz et al., 2004; Kim J.M. et al., 2012) and embryogenesis (Wang et al., 2007). Interestingly, a *mpk6* mutant transformed with a kinase-dead form of MPK6 has a very similar root phenotype to *yda1* plants. This indicates that MPK6 acts downstream of YODA in an auxin-dependent manner to control cell division in post-embryonic root development (Smekalova et al., 2014).

The recent work Enders et al. (2017) provided evidence that the AtMKK3-MAPK1-RBK1 (ROP binding protein kinase 1) module regulates auxin dependent cell expansion in Arabidopsis via modulation of the Rho-like GTPase (ROP4 and ROP6) activity. Both *mpk1* and *mkk3*-1 mutants display similar phenotypes to the effects of the auxins on the inhibition of root elongation and cotyledon expansion, suggesting that the MKK3-MPK1 pathway negatively regulates auxin-dependent cell growth. Strikingly, the known upstream MKK3 activators, MAPKKK17/18, which are clearly involved in ABA signaling (Danquah et al., 2015; Matsuoka et al., 2015; Mitula et al., 2015), were not investigated. Future studies should consider following up on this preliminary result in order to investigate the role of the ABA-activated MAPKKK17/18-MKK3-MPK1/2/7/14 module in the crosstalk between ABA and auxin signaling.

Overall, this section of the review highlights exciting results that suggest a connection between MAPK modules and the auxin signaling pathway. Disappointingly, though, current knowledge of MAPK cascade involvement in auxin-mediated processes is still fragmentary and no complete MAPK module has been confirmed as having a role in auxin signaling. Thus, multiple challenges and unanswered questions remain to be addressed.

## MAPKs in Jasmonic Acid and Salicilic Acid Signaling

Jasmonic acid and salicylic acid are plant hormones that participate in plant growth and development. JA plays essential roles in both biotic and abiotic stress responses (Heil et al., 2012; Hou et al., 2013; Wasternack and Hause, 2013). Plant defenses against pathogens are also mediated by SA, a type of phenolic acid, which similarly to JA also plays a role in plant growth and development (Hayat and Ahmad, 2007). In addition to its involvement in the response to wounding, SA participates in systemic acquired resistance (SAR) and the responses to abiotic stresses such as water, salinity and cold stress (Miura and Tada, 2014; Wendehenne et al., 2014). MAPKs are clearly involved in both signaling pathways as both positive and negative regulators. However, much work is still needed to elucidate downstream MAPK targets involved in SA- and JA-dependent processes.

### MAPKs in JA Signaling

**FIGURE 2:**
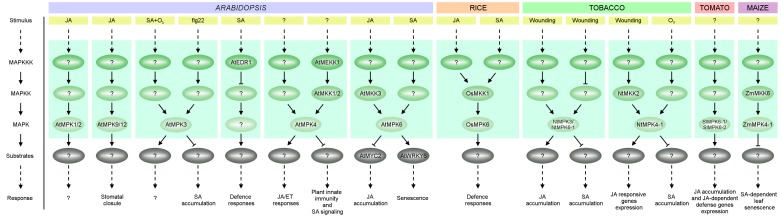
A simplified overview of MAPK cascades involved in JA and SA signaling in plant species such as: *A. thaliana* (At), *O. sativa* (Os), *Nicotiana tabacum* (Nt), *S. lycopersicum* (Sl) and *Z. mays* (Zm). Activation of MAPKs by various stimuli causes phosphorylation of MAPK effectors (usually transcription factors) further triggering cellular responses. See text for details.

Plant hormone JA is an important regulator of plant growth and development, but it plays a more important role in the wounding response and SAR (Heil et al., 2012; Hou et al., 2013; Wasternack and Hause, 2013). Despite the fact that crosstalk between JA and MAPK signaling has been reported, only a few studies have summarized this interaction. Nevertheless, MAPKs are reported to regulate JA biosynthesis and the expression of JA-dependent genes. For example, tomato SlMPK6-1 (also known as SlMAPK2, LeMPK2 and SlMPK2) and SlMPK6-2 (SlMAPK1, LeMPK1, SlMPK1) function as positive regulators of JA biosynthesis and signaling pathways (Kandoth et al., 2007). Simultaneous silencing of *SlMPK6-1* and *SlMPK6-2* has been shown to reduce JA biosynthesis and the expression of JA-dependent defense genes (**Figure 2**). On the other hand, JA regulates both MAPK activity and *MAPK* gene expression. In Arabidopsis, induction of AtMPK1/2 kinase activity is observed in leaves 1 h after JA treatment (**Figure 2**) (Ortiz-Masia et al., 2007). Furthermore AtMPK9 and AtMPK12 together are involved in JA-induced stomatal closure (Khokon et al., 2015; de Zelicourt et al., 2016; Lee et al., 2016) (**Figure 2**). Induction by JA treatment, albeit only at the transcript level, has also been demonstrated in rice for *OsMPK7*, *OsMPK20-5* and *OsMPK16* (Reyna and Yang, 2006; Singh and Jwa, 2013). Increased transcript levels after JA treatment have been observed for *BnaRaf30* in canola (Sun et al., 2014), for *Cucumis sativus MPK6* (*CsMPK6*), *CsMPK9-1, CsMPK20-1, CsMPK20-2, CsMKK4, CsMKK6*, and *CsMEKK21-1* in cucumber (Wang J. et al., 2015), for *NtMPK1, NtMPK7, NtMPK22-1* (also known as NtMPK16) and *NtMPK22-2* (also known as NtMPK17) in tobacco (Zhang et al., 2013), for 23 MAPKs in cotton (*Gossypium raimondii*) including *GrMPK2/3/5-1/18-20/22-25/27-28* (Zhang et al., 2014e), and for *Brachypodium distachyon MPK7-1* (*BdMPK7-1*) and *BdMPK20-5* in purple false brome (Jiang et al., 2015). Undoubtedly much work is still needed to fully resolve the potential roles of MAPKs in JA biosynthesis, JA signaling and JA-mediated responses and these pathways are attractive targets for future research.

### MAPK-Dependent SA Signaling

Alongside JA, SA – a type of phenolic acid – also plays an important role in plant growth, development and defense (Hayat and Ahmad, 2007). In addition to its involvement in the response to wounding, SA participates in SAR and responses to abiotic stresses such as water, salinity, and cold stress (Miura and Tada, 2014; Wendehenne et al., 2014). The activity of Arabidopsis AtMPK3 and AtEDR1, and maize ZmMKK6-ZmMPK4-1, are known to be regulated by SA (**Figure 2**). Thus, AtMPK3 has been shown to be induced by ozone stress in an SA-dependent manner (Ahlfors et al., 2004; Samajova et al., 2013). In disease resistance, MPK3 seems to be an important crosstalk regulator of late immune responses (Ichimura et al., 2006; Han et al., 2010; Mao et al., 2011; Meng and Zhang, 2013). Besides its well-known role in repressing the constitutive and flg22-induced expression of defense genes (Asai et al., 2002; Galletti et al., 2011; Montillet et al., 2013), AtMPK3 also appears to be a negative regulator of flg22-induced SA accumulation (Frei Dit et al., 2014).

Other studies have revealed that SA-inducible defense responses are also negatively regulated by the Raf-like MAPKKK, AtEDR1, indicating that AtEDR1 is involved in SA signaling, but not in JA/ET signaling (Frye and Innes, 1998; Frye et al., 2001; Virk et al., 2015). Recent studies also suggest the participation of ZmMKK6 in SA signaling: expression of inactive ZmMKK6 in Arabidopsis transgenic plants induced SA accumulation and SA-dependent leaf senescence. ZmMKK6 also activates both ZmMPK4-1 (also called ZmMPK4, ZmSIMK) and AtMPK4 *in vitro*. These data indicate that the ZmMKK6-ZmMPK4-1 cascade may play an important role in the regulation of SA-dependent leaf senescence (Li et al., 2016).

Analysis of expression profiles of MAPK cascade kinases after treatment with SA also led to the identification of kinases that might be involved in SA signaling in other species. In tomato, three out of five known MAPKK genes (*SlMAPKK1/2/4*), almost half of the MEKK subfamily genes, nearly half of the RAF subfamily genes and nearly all the ZIK subfamily genes were significantly upregulated by SA treatment (Wu et al., 2014). Transcription of the genes encoding AtRaf43 in Arabidopsis (Virk et al., 2015), BdMPK3 and BdMPK17 in purple false brome (Jiang et al., 2015), BnaMAPKKK18, BnaRaf28, BnaMKK1-2, BnaMKK4, BnaMKK9, BnaMPK1, BnaMPK3, BnaMPK5, BnaMPK6, BnaMPK19 in canola (Liang et al., 2013; Sun et al., 2014), GrMPK2/3/5/6/7/8/9/12/13/16/18/22/23/25/28 in cotton (Zhang et al., 2014e), NtMPK9-2 and NtMPK15 in tobacco (Zhang et al., 2013), OsMPK17-1 and OsMPK17-2 in rice (Hamel et al., 2006; Singh and Jwa, 2013), SlMKK3 in tomato (Li X. et al., 2014), and PsMAPK3 in pea (Barba-Espín et al., 2011) has also been shown to be significantly increased after treatment with SA. Future research is needed to identify novel components and effectors of these SA-dependent MAPK pathways.

### Crosstalk Between MAPK Cascade Kinases and Both JA and SA Signaling – An Insight Into Plant Immunity

MAPKs are clearly involved in plant defense signaling. Certain MAPKs such as AtMPK4 and AtMPK6 in Arabidopsis are involved in both JA and SA signaling (**Figure 2**). AtMPK4 positively regulates JA/ET responses (Brodersen et al., 2006), whereas in the AtMEKK1–AtMKK1/2–AtMPK4 cascade appears to function as a negative regulator of plant innate immunity and SA signaling (Petersen et al., 2000; Andreasson et al., 2005; Brader et al., 2007; Pitzschke et al., 2009). AtMPK6 participates in SA-induced detached leaf senescence by promotion of AtNPR1 activation (Chai et al., 2014). On the other hand, activation of the AtMKK3–AtMPK6 cascade in Arabidopsi*s* plants by JA represses a positive regulator of JA biosynthesis genes (AtMYC2), leading to suppression of JA production (Takahashi et al., 2007). In tobacco, NtMPK3 (also known as NtWIPK, NtMPK5) and NtMPK6-1 (NtSIPK, NtMPK6) appear to play an important role in wound-induced biosynthesis of JA and they function as repressors of SA accumulation in response to wounding (Seo et al., 2007; Oka et al., 2013; Hettenhausen et al., 2014). In addition to NtMPK3 and NtMPK6-1, another wounding-activated MAPK, NtMPK4-1 (NtMPK4, NtMPK1) appears to positively regulate JA signaling pathways and is also involved in SA signaling by affecting SA biosynthesis and signaling in response to ozone exposure (Gomi et al., 2005) (**Figure 2**). The induction of other *Nicotiana* MAPKs, such as NtMPK16 (NtMPK10) and NtMPK20 (NtMPK8), at the transcript level in response to MeJA and SA treatment may suggest that these kinases also play a role in JA and SA signaling, but further studies are needed to confirm this (Zhang et al., 2013). The putative involvement of MAPKs in both JA and SA signaling has also been reported in other species, such as *O. sativa*, *Z. mays*, and *S. lycopersicum*. In rice, the kinase activity of OsMPK17-1 *(OsMPK12, OsBWMK1)* is activated by both JA and SA treatment (Cheong et al., 2003; Singh and Jwa, 2013). Overexpression of OsMPK17-1 in tobacco causes SA and H_2_O_2_ accumulation and elevated *PR* gene expression, leading to hypersensitive response (HR)-like cell death (Cheong et al., 2003; Bigeard and Hirt, 2018). *OsMKK1* (*OsMEK2*), as well as *OsMPK6* (also called OsMPK1, OsMAPK6 and *O. sativa* SA-induced protein kinase – OsSIPK), *OsMPK17-1* and *OsMPK3* (also called *OsMPK5*, *OsMAP1*, *OsMAPK2*, *OsMSRMK2* and *OsBIMK1*), are transcriptionally induced by both JA and SA treatment in rice. In addition, overexpression of OsMPK6 results in JA and SA accumulation when challenged by pathogens, indicating that the OsMKK1-OsMPK6 cascade may be involved in JA- and SA-inducible defense responses (Singh and Jwa, 2013) (**Figure 2**). In maize, the MAPK ZmMPK3-2 (ZmMPK3) is sensitive to various signaling molecules, including JA or SA (Wang X.J. et al., 2010; Smekalova et al., 2013). In tomato, SlMKK4 (SlMKK2) and SlMKK9 (SlMKK4) seem to be involved in both JA and SA signaling pathways (Li X. et al., 2014). SA and JA signaling are crucial to plant defense against pathogens. Many examples of MAPK cascade kinases involved more or less directly in JA, SA or both JA and SA signaling demonstrate the importance of this cooperation for plants in response to wounding. However, there is still very little known about the details of this cooperation and further studies are needed to understand how this leads to improved resistance of plants to pathogens.

## MAPK Modules Involved in Brassinosteroid Signaling

**FIGURE 3:**
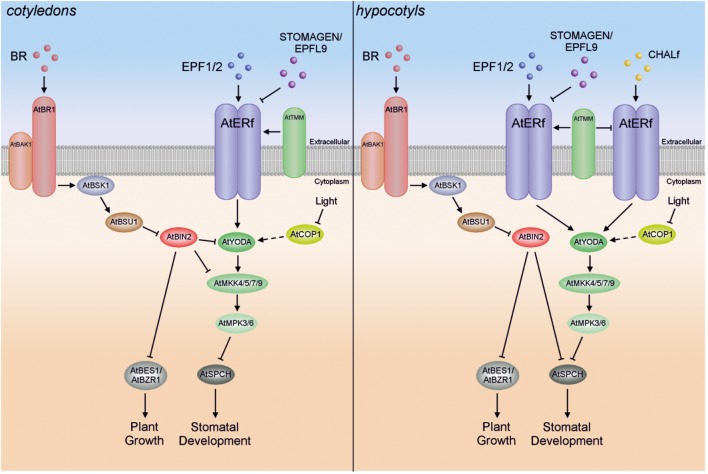
Schematic illustration of the GSK3-like kinase AtBIN2-mediated crosstalk between the AtYODA-AtMKK4/5-AtMPK3/6 cascade and BR signaling in cotyledons and in hypocotyls.

Relatively recent studies have shown that crosstalk also exists between Arabidopsis MAPK cascade kinases and a class of polyhydroxylated steroid hormones, the BR (Kim T.W. et al., 2012; Kang et al., 2015). In particular, BRs participate in cell division and cell elongation, but also take part in cellular patterning (Tang et al., 2011; Khan et al., 2013) (**Figure 3**). It was demonstrated that BRs repress stomatal development in cotyledons, but in an AtBZR1-independent fashion (Kim T.W. et al., 2012; Serna, 2013). The signal transduction required for correct stomatal patterning is mediated by the Arabidopsis ERECTA family (AtERf) of receptor-like kinases and the AtYODA-AtMKK4/5-AtMPK3/6 cascade, which results in phosphorylation and thereby inactivation of transcription factors such as Arabidopsis speechless (AtSPCH), AtMUTE and AtFAMA (Lau and Bergmann, 2012; Kim T.W. et al., 2012; Le et al., 2014). Recent studies have shown that phosphorylation of AtSPCH Serine 186 (one of three primary phosphorylation targets) plays a crucial role in stomatal formation (Yang et al., 2015). To summarize, when the BR level is high, BR signal transduction through plasma-membrane receptor brassinosteroid-insensitive 1 (AtBRI1), BR-signaling kinase 1 (AtBSK1) and phosphatase AtBRI1 suppressor 1 (AtBSU1) inactivates the glycogen synthase kinase 3 (GSK3)-like kinase BR insensitive 2 (AtBIN2), making AtBES1 (BRI1-EMS-suppressor 1; also called brassinazole-resistant 2 – BZR2)/AtBZR1, and the MAPK cascade (repressing AtSPCH) active, which in turn leads to promotion of plant growth and inhibition of cell division and stomatal formation in cotyledons, respectively (Kim T.W. et al., 2012; Le et al., 2014; Zhang et al., 2014a). On the other hand, when BR levels are low, AtBIN2 remains active, inhibiting AtBES1/AtBZR1 and the AtYODA-AtMKK4/5-AtMPK3/6 module, and leading to inhibition of plant growth and promotion of stomatal development in cotyledons (**Figure 3**) (Kim T.W. et al., 2012). It has also been demonstrated in two different studies by *in vitro* and/or yeast two-hybrid assays that AtBIN2 seems to inhibit the AtYODA-AtMKK4/5-AtMPK3/6 cascade by direct suppression of both AtYODA (MAPKKK) and AtMKK4/5 (Kim T.W. et al., 2012; Khan et al., 2013; Le et al., 2014; Xu and Zhang, 2015).

In contrast to their action in cotyledons, BRs seem to promote stomatal development in the hypocotyl, but in a BES1- and BZR1-independent manner (Serna, 2013). Interestingly, AtSPCH seems also to be controlled by AtBIN2, which phosphorylates the same AtSPCH residues as AtMPK3/6 (Gudesblat et al., 2012). Since AtYODA-AtMKK4/5-AtMPK3/6 cascade activity is likely reduced by CHALLAH family (CHALf) signaling in the hypocotyl, meaning that AtSPCH is not inhibited by this pathway, inactivation of AtSPCH by AtBIN2 might be the predominant pathway in hypocotyls (Le et al., 2014). Thus, in the hypocotyl, BR signaling inactivates AtBIN2 at high BR levels, whereas AtBES1/AtBZR1 and AtSPCH remain active, leading to promotion of plant growth, cell division and stomatal formation. Conversely, AtBIN2 remains active at low BR levels and then inhibits AtBES1/AtBZR1 and AtSPCH, resulting in inhibition of plant growth and promotion of stomatal development in hypocotyls (**Figure 3**).

Recent studies have also identified crosstalk between MAPK cascade kinases and BR signaling pathways in other species. OsMKK4 seems to be involved in BR signaling pathways (Duan et al., 2014) and might be involved in BR signaling in a similar manner to AtMKK4, because one of the rice orthologs of AtBIN2, OsGSK2, is involved in BR signaling (Tong et al., 2012). In tomato, all the MAPKs of group A are involved in BR-induced nematode resistance (Song et al., 2018), two of which, SlMPK6-1 (SlMPK2) and SlMPK6-2 (SlMPK1), positively regulate BR-induced pesticide metabolism (Yin et al., 2016). Only SlMPK6-1 plays a role in the regulation of BR-induced H_2_O_2_ accumulation and tolerance to oxidative and heat stress (Nie et al., 2013). In maize, the homolog of AtMPK6, ZmMPK6-2 (also called ZmMPK5), is involved in BR signaling. ZmMPK6-2 is activated by BR-induced H_2_O_2_ accumulation and in turn enhances apoplastic H_2_O_2_ accumulation via gene expression of NADPH, leading to up-regulation of antioxidant defense systems in leaves (Zhang et al., 2010). The significant involvement of MPK3 or/and MPK6 in BR signaling in different species might suggest that the crosstalk between MAPK and BR is evolutionarily conserved. However, in chinese cabbage (*Brassica rapa*), has the investigation of MAPK expression profiles revealed that five other genes are induced after BR treatment (Lu et al., 2015): *Brassica rapa* MAPK5 (BraMAPK5), BraMAPK17-1, BraMAPK17-2, BraMAPK18-1 and BraMAPK19-1.

## MAPK Kinase Cascades in Ethylene Biosynthesis and Signaling

**FIGURE 4:**
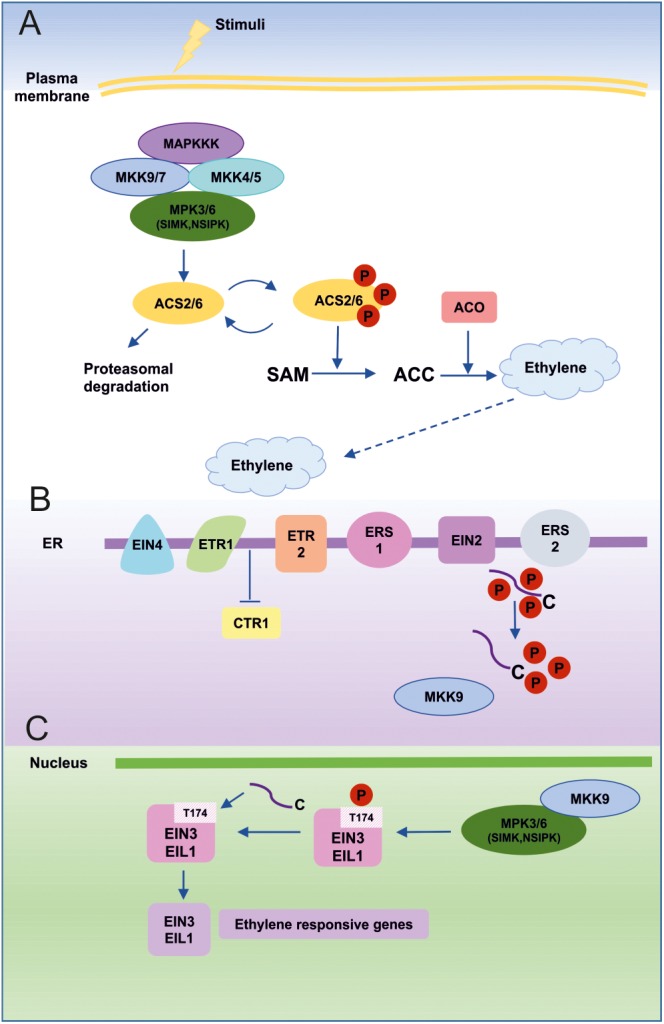
MAPKs in ET biosynthesis and signaling. **(A)** An external stimulus leads to activation of ET biosynthesis predominantly through the MKK9-MPK3/MPK6 cascade. Alternatively, MKK7 may also be involved in MPK3/MPK6 activation due to similarities with the MKK9 sequence and its activation mechanisms. MPK3 and MPK6 can also be activated by MKK4 and MKK5, which act in a redundant fashion upstream of MPK3/MPK6, especially after wounding-induced ET biosynthesis. SIMK (MsMPK6) and NSIPK(NtMPK6-1) are homologs of AtMPK6 from alfalfa and tobacco, respectively. Active MPK6 phosphorylates ACS2/ACS6, which initiates ET biosynthesis. **(B)** ET is perceived by five different receptors (ETR1, ETR2, ERS1, ERS2, EIN4) localized in the endoplasmic reticulum (ER) membrane and this leads to inhibition of CTR1 kinase activity, which is the primary negative regulator of ET signaling. As a consequence, MKK9 is released from CTR1 inhibition and translocates to the nucleus, where it activates MPK3 and MPK6. Moreover, inactive CTR1 is no longer able to phosphorylate the C-terminal domain (CEND) of EIN2. Dephosphorylated CEND moves to the nucleus and takes part in EIN3 stabilization. **(C)** In the nucleus, active MPK3/MK6 promotes the stability of the main plant-specific ET-dependent transcription factors (EIN3 and EIL1). Phosphorylation of EIN3 at the T174 position blocks its proteasomal degradation and enables it to activate ET-responsive genes.

MAPKs are also involved in ET biosynthesis and signaling. ET is a gaseous hormone involved in many aspects of plant biology, such as germination, plant growth, organ senescence and fruit ripening (Yang and Hoffman, 1984; Bleecker and Kende, 2000; Skottke et al., 2011; Dubois et al., 2018). Furthermore, it integrates external and internal signals to provide a dynamic response to diverse stress conditions (Yoo et al., 2009). ET sensing and signal transduction in plants are complex processes (**Figure 4**).

### MAPKs in the Regulation of Ethylene Biosynthesis

As shown in many studies, the MKK9-MPK3/MPK6 cascade is involved in the regulation of ET biosynthesis (Liu and Zhang, 2004; Joo et al., 2008; Xu J. et al., 2008; Skottke et al., 2011). The basal ET level is very low, but under special conditions (abiotic stress, wounding, pathogen infection, nutrient availability) ET production increases dramatically (Zarembinski and Theologis, 1994; Wang et al., 2002; De Paepe and Van der Straeten, 2005; Stepanova and Alonso, 2009; Iqbal et al., 2013; Ludwików et al., 2014; Tao et al., 2015; Chardin et al., 2017). Key enzymes in ET biosynthesis are ACC synthases (ACS; 1-aminocyclopropane-1-carboxylate synthases), which are strictly regulated at both the transcriptional and post-translational levels, (Kende, 1993).

Kim et al. (2003) showed that NtMPK6-1 is able to induce ET biosynthesis. The authors constructed transgenic plants overexpressing a constitutively active mutant of *NtMKK4*^DD^ (*NtMEK2*^DD^ T227D/S233D; kinase upstream of NtMPK6-1; Yang et al., 2001) under the control of a steroid-inducible promoter. Dexamethasone treatment resulted in immediate NtMPK6-1 activation and significant elevation of ET production. In experiments with transgenic Arabidopsis plants that overexpress *NtMKK4*^DD^, *AtMKK4*^DD^, and *AtMKK5*^DD^ under the control of the same steroid-inducible promoter, Liu and Zhang (2004) showed that MAPK activation mechanisms are conserved between species. Thus, NtMKK4^DD^ is able to activate MPK6/MPK3 in Arabidopsis. Analogously, Arabidopsis MKK4^DD^ and MKK5^DD^ (AtMKK4 and AtMKK5 are functional orthologs of NtMKK4^DD^) can activate the endogenous NtMPK3/NtMPK6-1 in tobacco plants. Further experiments demonstrated that MPK6 is essential for NtMKK4^DD^-dependent ET biosynthesis.

Analysis of known Arabidopsis ACS protein sequences revealed potential MAPK phosphorylation sites in ACS1, ACS2, and ACS6 (**Figure 4**). All three ACC synthases cluster together on a phylogenetic tree (Chae et al., 2003; Yamagami et al., 2003). An in-gel kinase assay confirmed that MPK6 is responsible for ACS6 phosphorylation. Site-directed mutagenesis showed that MPK6 is able to phosphorylate three serines (S480, S483, S488) in the ACS6 sequence. However, experiments with wild-type and mutated forms of ACS6 in which single, double and triple serine (S) residues were converted to alanine (A) or aspartic acid (D) revealed that changes in phosphorylation state do not alter its enzyme activity. Instead, it was suggested by Liu and Zhang (2004) that MPK6-mediated phosphorylation may influence ACS6 and ACS2 stability. Indeed, it turns out that mutated ACS6^DDD^, which mimics the phosphorylated state, is much more stable in transgenic plants than wild-type ACS6 and ACS6^AAA^. MPK6 phosphorylation sites are localized within the C-terminal domain of ACS6 and ACS2, which is the regulatory domain responsible for their stability. Lack of MPK6-mediated phosphorylation results in decreased ACS6 and ACS2 stability and immediate targeting of both proteins for proteasomal degradation. Dephosphorylation by ABI1, a member of the protein phosphatase 2C (PP2C) family is also involved in regulating the proteasomal degradation of ACS6 (Ludwików et al., 2014; Ludwików, 2015).

MAPKs regulate ET biosynthesis by controlling transcription of ACS. Recent studies showed that wounding-induced ET biosynthesis in Arabidopis is also under the control of MAPKs (Li et al., 2018). Analysis of ET accumulation after wounding in single *mpk3* and *mpk6* mutants and in a double *mpk3 mpk6* mutant rescued by MPK3^TA^ or MPK6^YG^ (chemically synthesized MPK3 and MPK6) revealed that MPK6 is the dominant kinase in this process (Xu et al., 2014, 2016). Li C.H. et al. (2014) observed a 50% reduction in wounding-triggered ET accumulation in the *mkk6* mutant compared to control plants. In many developmental processes, kinases MKK4 and MKK5 are redundant and function upstream of MPK3/MPK6 (Xu and Zhang, 2015), as seen in the case of wounding-induced ET accumulation. Thus, in a single *mkk4* mutant, ET biosynthesis was reduced by about 10% compared to wild-type plants, while in a *mkk5* single mutant, the reduction in ET accumulation was about 50%. However, in a double mutant (*mkk4 mkk5*) strain, ET production was reduced by 80%. Therefore, MKK4 and MKK5 act upstream of MPK3 and MPK6 after wounding and are required for wounding-dependent ET accumulation. Among the family of ACC synthase genes, only four are induced after wounding, namely *ACS2, ACS6, ACS7*, and *ACS8*. Genetic studies confirmed that changes in expression of these four ACS genes are under the control of MKK4 and MKK5. In double *mkk4 mkk5* mutant plants, *ACS2, ACS6, ACS7*, and *ACS8* expression was reduced. Analysis of the role of downstream elements of the MAPK cascade showed that, in *mpk6* single mutant plants, the expression level of all four ACC synthase genes was markedly reduced, but in *mpk3* single mutant plants was unaffected. Moreover, *ACS2, ACS6, ACS7*, and *ACS8* are activated at different times after wounding stimuli. *ACS6* and *ACS7* were induced very quickly, about 30 min post-wounding, while *ACS2* and *ACS8* expression reached a maximum around 2–6 h after wounding (Li et al., 2018). These data show that MAPKs are indeed involved in ET biosynthesis under wounding conditions. MAPKs likely influence the expression level of a subset of ACS genes, and thereby modulate ET biosynthesis, by activation of WRKY33 TFs (Li et al., 2012).

### MAPKs in Ethylene Signaling

Extensive research in Arabidopsis led to the identification of key elements of the ET signaling cascade (**Figure 4**). After revealing that CTR1 does not function as a MAPK cascade element, but instead inactivates EIN2 by direct phosphorylation of specific residues, extensive efforts have been made to identify MAPK cascades involved in ET signaling (Ju et al., 2012; Qiao et al., 2012; Wen et al., 2012; Cho and Yoo, 2015). Novikova et al. (2000) showed that a protein extract prepared from wild-type Arabidopsis plants treated with ET contained MAPK activity and that this activity was higher in *ctr1* (knockout) plants and lower in *etr1* (ET-insensitive) mutant plants than in wild-type. Immunoprecipitation experiments with the Arabidopsis extracts, using antibodies specific for the mammalian MAPK ERK1, identified a putative MAPK with molecular mass of 47 kDa (Novikova et al., 2000). Later, in 2003, Hirt’s group found the MAPKs MPK6 and MPK13 to be involved in ET signaling (Ouaked et al., 2003). They isolated protein extracts from *Medicago* and Arabidopsis cells before and after 1-aminocyclopropane-1-carboxylic acid (ACC) treatment and used these to perform in-gel kinase assays. As a result, they discovered two protein kinases 46 and 44 kDa in size in *Medicago*. These experiments led to the identification of strong kinase activity associated with SIMK (46 kDa) and MMK3 (44 kDa). The researchers also noticed that an increase in kinase activity did not correlate with an increased amount of these proteins, suggesting that ET induced MAPK activation by post-translational modification. SIMK from *Medicago* was most similar to MPK6 from Arabidopsis, while MMK3 corresponded to MPK13. Moreover, Ouaked and coworkers showed that MPK6 is constitutively active in a *ctr1* mutant and ET-dependent activation is not connected with EIN2 or EIN3. In Arabidopsis plants overexpressing *Medicago* MKK4 (MsSIMKK, MsSIMK kinase), they observed a *ctr1*-like phenotype in etiolated seedlings (Ouaked et al., 2003).

Yoo et al. (2008) demonstrated that the MKK9-MPK3/MPK6 cascade is involved in not only in ET biosynthesis, but also in ET signaling, acting downstream of CTR1 (**Figure 4**). It was also shown that MKK7 and MKK9 are able to activate both MPK3 and MPK6, which play a similar role in ET signaling (Novikova et al., 2000; Ouaked et al., 2003). What is more, ET-dependent activation of MPK3/6 by MKK9 is abolished in a *mkk9* mutant. The same authors also showed that overexpression of MKK7 and MKK9 in *ctr1* protoplasts results in specific activation of MPK3 and MPK6. These observations led to placement of the MKK9-MPK3/MPK6 cascade downstream of the key negative regulator of ET signaling, CTR1. Despite the fact that MKK7 and MKK9 are very similar in both sequence and mechanism of activation, the basal transcript level of MKK9 in protoplasts and in leaves is significantly higher than that of MKK7. Thus, MKK9 is considered predominant in ET signaling. Yoo et al. (2008) also investigated a positive role of MKK9 in ET signaling. Under most of the conditions examined, *mkk9* mutants present phenotypes similar to those of *ein3* mutants. Furthermore, in *mkk9* and *ein3* mutants, the expression of early ET signaling genes (*ERF1* and *ERF5*) in leaves is abolished. *ERF1* and *ERF5* are indirect targets of EIN3, the main TF regulating ET-inducible genes (Solano et al., 1998; Yanagisawa et al., 2003). Overexpression of permanently active MKK9 (MKK9a) results in constitutive ET signaling, which cannot be blocked by ET receptor mutants (*etr1*) or treatment with Ag^+^, an inhibitor of ET perception. These data support the assumption that MKK9-MPK3/6 functions downstream of CTR1 (Yoo et al., 2008). Perhaps even more interestingly, MKK9 acts a linker between CTR1, which is located in the ER, and other components of the ET signaling cascade located in the nucleus. After ACC treatment, MKK9 is able to move to the nucleus and activate MPK3 and MPK6, which are localized in both the nucleus and cytoplasm. After activation by MKK9, MPK3 and/or MPK6 phosphorylate(s) EIN3 in the nucleus. Computational analysis predicts two MAPK phosphorylation sites in the EIN3 protein. Mutation experiments reveal that phosphorylation at T174 is mediated by MPK6 and results in enhanced EIN3 stability (Alonso et al., 2003; Binder et al., 2007). However, after phosphorylation at the second MAPK phosphorylation site (T592), the stability of EIN3 is reduced. These findings show that the MKK9-MPK3/MPK6 cascade is the key module responsible for EIN3 stability and ET signaling (Yoo et al., 2008).

Results from different studies provide evidence for ET-dependent MKK9-MAPK3/6 activation, but there is still some controversy about the proposed model. It is worth noting that the MKK9-MAPK3/6 cascade is readily activated by environmental stresses (such as wounding and touch) (Alzwiy and Morris, 2007). It is even possible to activate the MAPK cascade mechanically by spraying “treatment” instead of ET or ACC treatment (Colcombet and Hirt, 2008). The involvement of the MKK9-MAPK3/6 cascade in ET signaling therefore needs to be scrutinized by precisely controlled experiments.

It is well known that ET signaling is an indispensable element of the response to various stimuli (salt stress, pathogen attack, iron deficiency or dehydration) (Kende, 1993; Xu J. et al., 2008; Han et al., 2010; Kazan, 2015; Tao et al., 2015; Ye et al., 2015; Chen J. et al., 2017; Khan et al., 2017). Salt stress stimulates ET biosynthesis which in turn activates other internal signals (Wang et al., 2002; Xu J. et al., 2008; Dong et al., 2011). However, the mechanisms by which the external signals relating to salinity stress stimulate ET biosynthesis remain unknown. Recent studies in *O. sativa* report that one of the receptor-like kinases (RLKs) involved in salt stress tolerance is able to phosphorylate both MPK3 and MPK6 (Ouyang et al., 2010; Li C.H. et al., 2014). RLKs are thought to be involved in transducing external signals into the cell, and some of the large number of known RLKs in *O. sativa* and *A. thaliana* are important in plant development (Osakabe et al., 2013) and the responses to drought and salinity stress (Marshall et al., 2012; Ouyang et al., 2010; Vaid et al., 2013). Salt Intolerance 1 (SIT1) is an active RLK that plays a significant role in drought and salt stress tolerance in *O. sativa*. *SIT1* is mainly expressed in root epidermal cells and its expression is induced immediately by NaCl. As a consequence, SIT1 activates MPK3 and MPK6 (Li C.H. et al., 2014). A co-immunoprecipitation assay showed that rice MPK3 and MPK6 are components of the SIT1 complex. What is more, *in vitro* phosphorylation experiments revealed that SIT1 is able to phosphorylate MPK3 and MPK6. A genetic approach confirmed that SIT1 acts upstream of MPK3 and MPK6 in *O. sativa*. It was also shown that SIT1 is involved in activation of antioxidant systems. ET signaling during plant stress responses is regulated by ROS production (Jung et al., 2009; Mergemann and Sauter, 2000). Li C.H. et al. (2014) demonstrated that SIT1-induced ROS accumulation requires ET production and signaling. Furthermore, they showed that after salinity-dependent activation, SIT1 is able to phosphorylate MPK3 and MPK6, resulting in salt sensitivity in rice. These findings are in line with other results showing that, when MPK3 and MPK6 are activated by MKK9, they increase salt sensitivity in Arabidopsis (Xu J. et al., 2008). There is a marked similarity between the rice SIT1-MPK3/MPK6 and Arabidopsis MKK9-MPK3/MPK6 cascades. However, whether SIT1 is involved in the rice MKK9-MPK3/MPK6 cascade needs to be examined.

Plants under attack by the necrotrophic fungal pathogen *Botrytis cinerea* produce high level of ET (Broekaert et al., 2006; van Loon et al., 2006), although precisely how ET biosynthesis is triggered by pathogen infection is still unclear. Han et al. (2010), using a double *mpk3 mpk6* mutant rescued by a DEX-inducible MPK6 cDNA construct, were able to show that, in response to *Botrytis cinerea*, the MPK3/MPK6 cascade is crucial for activating ET biosynthesis. These authors also identified ACC synthase 6 (ACS6) as the main enzyme contributing to *Botrytis cinerea*-induced ET production.

MPK3 and MPK6 are also involved in the regulation of ET biosynthesis during iron deficiency in Arabidopsis (Ye et al., 2015). Iron (Fe) is a vital microelement because Fe ions are a component of many of the enzymes controlling basic physiological processes including photosynthesis and chlorophyll biosynthesis (Kobayashi and Nishizawa, 2012). Ye et al. (2015) showed that lack of iron induces transcription of the *MPK3* and *MPK6* genes, as well as increasing MPK3 and MPK6 kinase activity. Moreover, the transcript levels of some ACC synthases are also increased in Fe-deficient plants. Although the regulation of Fe-induced ET biosynthesis needs further analysis, the work of Ye et al. (2015) highlights another mechanism involving the MPK3/MPK6 cascade.

MAPKs can be activated by many different stimuli. For example, in Arabidopsis seedlings, MPK6 may be activated by drought and rapidly inactivated during rehydration (MAPK Group, 2002; Tsugama et al., 2012; Xu and Chua, 2012). Interplay between dehydration and rehydration in plants is especially important for the cut-flower industry. Thus, rehydration after dehydration induces rapid ET production for a short duration in rose (*Rosa hybrida*) flowers (Tsugama et al., 2012). Further research on rose flowers showed that, particularly in the gynoecia, protein levels of RhMPK6 are high during both dehydration and rehydration, but RhMPK6 kinase activity was observed only within the first hour of rehydration. Active RhMPK6 is able to phosphorylate and stabilize RhACS1, stimulating ET production (Meng et al., 2014). The RhMPK6-RhACS1 module seems to be crucial for transduction of the rehydration signal and triggering of ET biosynthesis, which controls flower opening and senescence in rose. Rehydration-induced ET biosynthesis also seems to involve RhMKK9. *RhMKK9* is expressed 30 min after rehydration, but after 12 h the expression is almost undetectable. Chen and co-workers correlated these results for RhMKK9 with the expression and activity of RhMPK6 and RhACS1, and proposed that RhMKK9 functions as an activator of RhMPK6-RhACS1 (Chen J. et al., 2017). However, whether RhMKK9 is actually an upstream activator of RhMPK6 in dehydration-dependent ET biosynthesis in rose gynoecia must be confirmed by further experiments.

## MAPK Cascades in Abscisic Acid Signaling

**FIGURE 5:**
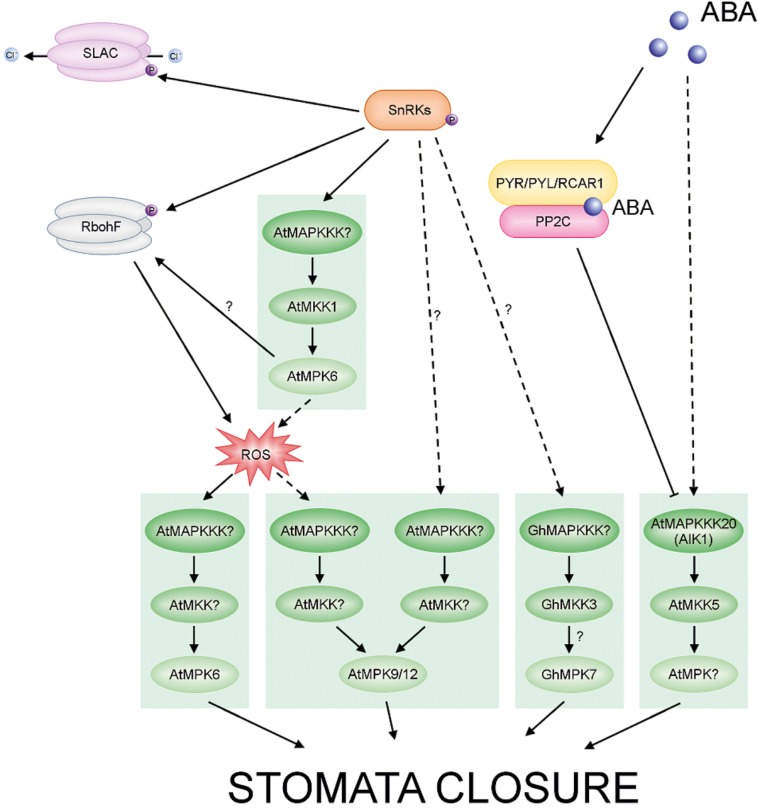
ABA-regulated MAPKs in Arabidopsis and cotton. ABA promotes stomatal closing. The different cascades are distinguished by different colors in the scheme. Arrows with solid lines represent established signaling pathways, while arrows with dashed lines represent putative signaling pathways. In the presence of ABA, PYR/PYL/RCAR receptors bind the phytohormone and inhibit group A PP2Cs. These events result in activation of SnRK2s. Activated SnRK2s phosphorylate and activate downstream targets, including MAPKs, Respiratory Burst Oxidase Homolog (RBOH) and Slowly Activating Anion Conductance (SLAC S-type). Active RBOH mediates ROS production. Note that in guard cells crosstalk between ABA signaling and ROS signaling may coincide at the MAPK level and regulates stomatal closure.

Abscisic acid signaling has been intensively studied and comprises multiple components including MAPKs. The plant hormone ABA functions as a key regulator in many developmental and physiological processes in plants, including seed dormancy and germination (Finkelstein et al., 2002; Nambara and Marion-Poll, 2003; Gutierrez et al., 2007; Chen M. et al., 2017; Née et al., 2017), seedling growth (Leon-Kloosterziel et al., 1996; Chen M. et al., 2017; Trupkin et al., 2017) and also adaptation to various biotic and abiotic stress conditions (Lee and Luan, 2012; Wang et al., 2018). Interestingly, the application of exogenous ABA to plant structures initiates the effect of stress conditions and results in transcriptional regulation, protein accumulation and activation of MAPKs, suggesting an important role for MAPK pathways in ABA signaling (Fujita et al., 2006; Xing et al., 2008; Zhang et al., 2014c,d; Li Y. et al., 2017, Li K. et al., 2017; Li Q. et al., 2017). Subsequent to binding of the hormone by different cellular receptors, ABA functions through a complex network of signal transduction pathways, which activate responses including the regulation of stomatal aperture and the expression of stress-responsive genes (Himmelbach et al., 2003; Leung and Giraudat, 1998; Finkelstein, 2013; Mitula et al., 2015; Albert et al., 2017; Eisenach et al., 2017). The core components of the ABA signaling pathway have been identified and characterized relatively recently (Fujii et al., 2009; Ma et al., 2009; Park et al., 2009). Initial steps in ABA signal transduction involve the PYR/PYL/RCAR ABA receptors and also the phosphatase/kinase enzyme pairs, PP2Cs and SnRK2s, respectively, which have antagonistic functions. The outcome of ABA signaling is the activation of gene expression by transcription factors under the control of SnRK2s (**Figure 5**) (Cutler et al., 2010; Klingler et al., 2010; Finkelstein, 2013; Nakashima and Yamaguchi-Shinozaki, 2013; Tan et al., 2018). These findings have certainly contributed to a more rapid understanding of the protein complexes that perceive and transmit ABA signals. Many previous studies indicate the participation of MAPK cascades in ABA-mediated responses, including antioxidant defense, guard cell signaling and seed germination (for reviews see also Liu, 2012; Danquah et al., 2014; Colcombet et al., 2016; de Zelicourt et al., 2016). Thus, the interactions between ABA signaling and other signaling pathways, including MAPK pathways, are beginning to be deciphered.

### MAPK Gene Transcription Regulated by ABA

In recent years, there has been a significant increase in research on ABA-mediated gene expression in a variety of plant species. ABA signal transduction pathways modulate gene expression, including changes in transcription levels, transcript processing and stability (Cutler et al., 2010). The regulation of ABA-responsive gene activity involves TFs, which recognize and bind to *cis*-elements in the promoter regions upstream of their target genes (Zhang et al., 2014b). Importantly, in addition to the action of TFs, ABA-responsive gene expression is mediated by receptors, secondary messengers and protein kinase/phosphatase cascades (Fujita et al., 2011). Nearly 10% of the protein-coding genes in *A. thaliana* are regulated by ABA, a far greater percentage than for other hormones (Shinozaki et al., 2003; Nakashima et al., 2009; Cutler et al., 2010; Fujita et al., 2011). Several Arabidopsis genes encoding particular members of the MAPK family have been reported to be transcriptionally regulated by ABA. These include *AtMPK1, AtMPK2* (Ortiz-Masia et al., 2007; Hwa and Yang, 2008; Umezawa et al., 2013), *AtMPK3* (Lu et al., 2002; Wang et al., 2011), *AtMPK5, AtMPK7* (Menges et al., 2008), *AtMPK18*, *AtMPK20* (Wang et al., 2011), *AtMKK9* (Menges et al., 2008), *AtMAPKKK1* (*ANP1*), *AtMAPKKK5* (Menges et al., 2008), *AtMAPKKK15* (Wang et al., 2011), *AtMAPKKK16* (Wang et al., 2011), *AtMAPKKK17*, *AtMAPKKK18* (Menges et al., 2008; Wang et al., 2011), *AtMAPKKK19* (Wang et al., 2011), *AtMAPKKK20* (Li K. et al., 2017), and *AtRaf6*, *AtRaf12*, and *AtRaf35* (Menges et al., 2008), all of which are regulated at the transcriptional level, indicating possible participation of these kinases in ABA signaling. It is worth mentioning that, despite the large number of ABA-regulated genes, the roles of most of them in ABA signaling have not been characterized. In searches for rice (*O. sativa*) MAPK genes transcriptionally activated by ABA, many genes were identified (**Supplementary Table 3**). It is worth mentioning that OsMPK3 (OsMPK5) is the best characterized of all the rice MAPKs, having been studied independently by at least six research groups and shown to be regulated by a variety of biotic and abiotic stresses (Agrawal et al., 2002; Huang et al., 2002; Song and Goodman, 2002; Wen et al., 2002; Reyna and Yang, 2006; Chen and Ronald, 2011; Nautiyal et al., 2013; Sharma et al., 2013; Jaemsaeng et al., 2018). Suppression of *OsMPK3 (OsMPK5)* by RNAi on the one hand results in reduced sensitivity to ABA, and on the other hand causes an increase in levels of endogenous ET (Xiong and Yang, 2003; Sharma et al., 2013). Many ABA-regulated genes have also been characterized in other plant species and these can be classified into two groups, upregulated and downregulated (**Supplementary Table 3**). The response of MAPK genes to ABA treatment suggest the involvement of these genes in ABA signaling. So far, the role of only a few of the kinases listed in **Supplementary Table 3** has been investigated in detail, and in the following sections the functional characterization of these kinase modules and the downstream responses they control is reviewed.

### MAPK Involvement in ABA Signaling in Guard Cells

**FIGURE 6:**
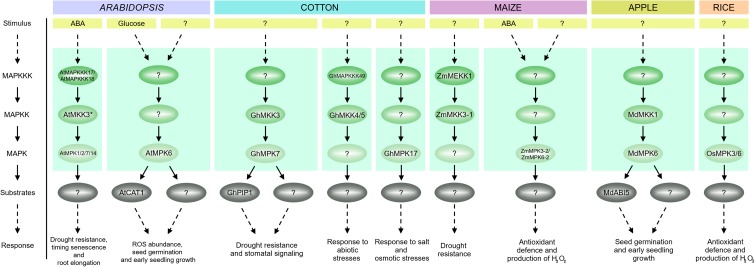
Overview of MAPKs regulated by ABA in different plant species. A single ABA-activated MAPK cascade MAPKKK17/18-MKK3-MPK1/2/7/14 has been identified in Arabidopsis. This pathway is involved in drought resistance, senescence, stomatal development and signaling. In addition, MKK3 in both maize and cotton has been shown to function in response to ABA. In maize, MKK3 acts downstream of MEKK1 and transcripts for both kinases are upregulated on ABA treatment. In cotton, ABA and drought induce activation of a MAPK cascade composed of MKK3, MPK7 and PIP1. These two pathways, MEKK1–MKK3 in maize and MKK3–MPK7–PIP1 in cotton, are associated with drought resistance and stomatal signaling. Another module in cotton, MAPKKK49-MKK4/MKK5, is involved in the ABA-mediated response to abiotic stress. MPK17 is another well-characterized MAPK in cotton, which regulates the response to salt and osmotic stresses. ABA-inducible genes encoding cotton MAPK cascade components presented in the scheme are *MKK3*, *MAP3K49* and *MPK17*, respectively. Some MAPK cascades have a similar function in different plant species. In Arabidopsis and apple, the MKK1-MPK6 module affects seed germination and early seedling growth. ABA treatment induces transcription of the genes encoding MKK1 and MPK6 in both plant species. In Arabidopsis, MKK1 mediates activation of MPK6, thereby regulating CATALASE1 expression in ROS homeostasis. Additionally, glucose treatment significantly increases MKK1 and MPK6 activities. In apple, ABA-responsive transcription factor ABI5 may act as a downstream target of this MAPK cascade. MPK5 and MPK3 in maize and MPK1 and MPK5 in rice are required for ABA-induced antioxidant defense and play a similar role to Arabidopsis MPK6. In maize, ABA treatment significantly increases MPK5 and MPK3 activities. In rice, ABA treatment induces *MPK1* and *MPK5* expression.

Abscisic acid is the main regulator of stomatal movement (Burnett et al., 2000; Dodd et al., 2003; Jiang and Song, 2008; Albert et al., 2017; Qu et al., 2018). The phytohormone may also cause the production of ROS in various plant cells or tissues (Hu et al., 2005; Zhang et al., 2011; Shang et al., 2016; Qi et al., 2017) and ABA signaling in guard cells is mediated by ROS (Jammes et al., 2009). Studies showing that MAPKs can be activated by ROS may indicate that ABA signaling and ROS signaling coincide at the MAPK level (Zhang et al., 2007), and crosstalk between these pathways could regulate stomatal closure. H_2_O_2_ is an another important signaling molecule in ABA-induced stomatal closure (Pei et al., 2000; Li Q. et al., 2017; Rodrigues et al., 2017). Thus, the generation of H_2_O_2_ in response to ABA results in a reduction in size of the stomatal aperture (Wang and Song, 2008; Li Q. et al., 2017). In *A. thaliana* MPK3 is involved in the perception of ABA and H_2_O_2_ in guard cells. The results of Gudesblat et al. (2007) indicate that MPK3 functions downstream of ROS in ABA inhibition of stomatal opening, but not in ABA-induced stomatal closure. Another study showed that the *atmkk1* and *atmpk6* mutants block ABA-dependent H_2_O_2_ production in guard cells (Xing et al., 2008) (**Figure 5**). In apparent contradiction of these results, Montillet et al. (2013) recently found that AtMPK3 and AtMPK6 are not involved in ABA-induced stomatal closure, but instead are involved in stomatal closure induced by biotic stress. The same authors confirmed, however, that ABA-induced stomatal closure is mediated by MPK9 and MPK12: *atmpk9 atmpk12* double mutants, but not single mutants, are impaired in ABA-induced stomatal closure, in ABA inhibition of stomatal opening, and in inhibition of the promotion of stomatal closure by H_2_O_2_ (**Figure 5**) (Jammes et al., 2009; Salam et al., 2012; de Zelicourt et al., 2016). Recently, Mitula et al. (2015) found an ABA-activated kinase MAPKKK18 to be involved in stomatal development and function. Under normal growth conditions, *mapkkk18* mutant plants show increased stomatal aperture and decreased abaxial stomatal index, compared to the wild-type. Moreover, Li Y. et al. (2017) demonstrated that the *mapkkk18* mutant displays impaired ABA-induced stomatal closure. The authors hypothesized that MAPKKK18 is probably involved in drought stress resistance by accelerating stomatal closing when drought stress occurs (Li Y. et al., 2017). Consistent with this, studies of the transcriptional regulation of the *MKKK18* promotor revealed high promoter activity following ABA stimulation in guard cells (Mitula et al., 2015). Importantly, experimental results indicate that MAPKKK18 interacts directly with two of the key proteins of the ABA core signaling module, PP2C phosphatase ABI1 (Mitula et al., 2015) and kinase SnRK2.6 (Tajdel et al., 2016). ABI1, in the absence of ABA, not only inhibits MAPKKK18 kinase activity by dephosphorylation, but also targets MAPKKK18 for degradation by the ubiquitin-proteasome pathway (UPS) (Ludwików, 2015; Mitula et al., 2015). However, when ABA binds to PYR/PYL receptors, MAPKKK18 degradation is blocked, and this stabilization allows the kinase to activate downstream components of the signaling module (Mitula et al., 2015). It is worth mentioning that recently two independent research groups reconstructed a complete MAPK cascade initiated by MAPKKK18 and regulated by ABA (Danquah et al., 2015; Matsuoka et al., 2015). These authors showed the ABA-regulated MAP3K17/18-MKK3-MPK1/2/7/14 cascade to be involved in stress signaling (Danquah et al., 2015) and timing of senescence (Matsuoka et al., 2015), and as previously mentioned it is also known to have a role in drought stress resistance (Li Y. et al., 2017) (**Figure 6**). Importantly, a close paralogue of MAPKKK18, MAPKKK17, was found in the Arabidopsis genome and was included in the study of Danquah et al. (2015). The kinase activity of both MAPKKK17 and MAPKKK18 is significantly increased after ABA treatment (Danquah et al., 2015; Mitula et al., 2015). In addition, there is a positive correlation between the transcription levels of ABA core signaling genes and the MAPKKK17/MAPKKK18 genes (Danquah et al., 2015).

Arabidopsis ABA-insensitive protein kinase 1 (AIK) is another MAPKKK, MAPKKK20, involved in the regulation of ABA-induced responses. Very recently, Li K. et al. (2017) documented that MAPKKK20 is a positive regulator of ABA-induced stomatal closure and also regulates the effect of ABA on root architecture. Arabidopsis *AIK* insertion mutants are insensitive to ABA and do not display stomatal closure and root elongation in response to ABA treatment. Moreover, the number of stomata in *aik1* mutants is greater than in wild-type plants. The authors also showed that, as in the case of MAPKKK18 (Mitula et al., 2015), MAPKKK20 is regulated by ABA at both transcript and protein levels. ABA induces AIK1 activity in Arabidopsis and tobacco and, significantly, this kinase activity is inhibited by ABI1, which dephosphorylates AIK. Finally, analysis of *mpk6* and *mkk5* single mutant plants showed them to have a similar phenotype to *aik1* single mutant plants and experiments using bimolecular fluorescence complementation demonstrated that AIK1 works upstream of MKK5-MPK6: MKK5 is phosphorylated and is thus activated by AIK1 in an ABA-activated process. From these findings, it is tempting to hypothesize that sequential phosphorylations of the AIK1 (MKKK20)-MKK5-MPK6 module are involved in ABA- mediated regulation of both the stomatal response and primary root growth (Li K. et al., 2017) (**Figure 5**).

### MAPKs Implicated in ABA Signaling During Seed Germination

In addition to stomatal closure, ABA has other important physiological effects relating to seed maturation and the inhibition of seed germination (Koornneef et al., 2002; Xing et al., 2009; Chiu et al., 2016; Devic and Roscoe, 2016; Huang et al., 2017; Leprince et al., 2017). Using ABA-mediated inhibition of germination as a selection criterion, a number of important players in ABA signaling, including MAPKs, have been discovered through genetic screens (Joseph et al., 2014). Other evidence indicates that MAPK cascades are positive regulators of ABA signaling during seed germination, when plants overexpressing AtMPK1 and AtMPK2 display hypersensitivity to ABA (Hwa and Yang, 2008). Interestingly, a phosphoproteomic study showed that SnRK2 promotes activation of AtMPK1 and AtMPK2 in an ABA-dependent manner (Umezawa et al., 2013). AtMKK3 has been suggested as the upstream activator of AtMPK1 and AtMPK2 (Hwa and Yang, 2008). Indeed, Danquah et al. (2015) reported that the MKK3-MPK1/2/7/14 module mediates ABA signaling during germination and root elongation. Thus, *mkk3-1* plants are hypersensitive to ABA during germination and root elongation, while the seeds of this mutant are hypersensitive to increasing ABA concentrations. Correspondingly, MKK3-overexpressing seeds were less sensitive to increasing ABA concentrations (Danquah et al., 2015). Importantly, MAPKKK18, which functions upstream of MKK3, is associated with the control of seed development and dormancy. Mitula et al. (2015) demonstrated that the germination of *mkkk18* knockout plant lines is inhibited in medium supplied with ABA. Taken together, these results suggest that the MAPKKK18-MKK3 module mediates ABA signaling during germination and root elongation.

The Raf10 and Raf11 kinases are also involved in regulating seed dormancy and the response to ABA, as they affect the expression of ABA-regulated genes (including *ABI3*, *ABI5*) (Lee et al., 2015). The above mentioned AtMKK1–AtMPK6 cascade is also involved in ABA signaling during seed germination. The single mutants *mkk1* and *mpk6*, as well as the *mkk1 mpk6* double mutant, all show insensitivity to ABA during germination, while plants overexpressing MKK1 and MPK6 are hypersensitive to ABA (Xing et al., 2007, 2008). Interestingly, in apple, the MdMKK1–MdMPK1 cascade has a similar function to AtMKK1–AtMPK6 in Arabidopsis. Expression of MdMKK1 and MdMPK1 results in ABA hypersensitivity during seed germination, implicating MdMKK1 and MdMPK1 in the positive regulation of ABA signaling during seed germination and early seedling growth (Wang X.J. et al., 2010) (**Figure 6**).

### ABA-Regulated MAPKs in Maize

So far, only a few members of the MAPK family have been identified and well documented in *Z. mays*. These include ZmMPK3-2 and ZmMPK6-2 (ZmMPK5), which are both activated by ABA-induced production of H_2_O_2_ and increase the tolerance of plants to drought, salt stress and oxidative stress (Wang J. et al., 2010). ZmMPK3 and ZmMPK6-2 play a similar role in ABA-induced antioxidant defense as AtMPK6 in Arabidopsis (Xing et al., 2008), and OsMPK3 (OsMPK5) and OsMPK6 (OsMPK1) in rice (Zhang H. et al., 2012; Shi et al., 2014). Interestingly, ZmCPK11, one of the calcium-dependent protein kinases (CDPKs), has been shown to act upstream of ZmMPK6-2 in ABA signaling in maize (Ding et al., 2013). Moreover, very recently the underlying molecular mechanisms have been elucidated. Ma et al. (2016), identified ZmABA2 as a protein interacting with ZmMPK6-2. ZmMPK6-2 phosphorylates ZmABA2, which results in an increase in ABA content. These findings show that ZmABA2 is a direct target of ZmMPK6-2 and participates in ABA biosynthesis and function.

Another study implicates the maize gene *ZmMKK3-1 (ZmMKK3)*, which encodes a MAPKK, in the ABA signal transduction pathway, since *ZmMKK3-1* is upregulated by ABA. Its overexpression on the one hand results in increased tolerance to osmotic and oxidative stresses, but on the other hand causes a decrease in ABA sensitivity in transgenic tobacco plants (Zhang M. et al., 2012). In maize root the expression of another MAPKK, *ZmMKK1*, is also induced by ABA. Overexpression of ZmMKK1 confers tolerance to salt and drought in Arabidopsis and yeast. ZmMKK1 interacts with ZmMEKK1 *in vitro*, and this, importantly, represents the first characterized MAPK cascade in maize (Cai et al., 2014). ABA has also been shown to induce transcription of other MAPKs in maize, including *ZmMPK4-1* (Wang et al., 2014), *ZmMPK7* (Zong et al., 2009), and *ZmMPK17* (Pan et al., 2012). ZmMPK7 together with ZmMPK3 is activated by ZmMKK10 (Chang et al., 2017).

### ABA-Regulated MAPKs in Other Species

The participation of MAPKs in ABA signaling has been best characterized in *A. thaliana*. Nevertheless, MAPKs are known to be involved in this signaling pathway in other species, as recent research has shown. In mulberry (*Morus* L.) expression of *MnMPK1* is upregulated by ABA (Liu et al., 2017). In pea (*P. sativum* L.), using a kinase activity assay, Ortiz-Masia et al. (2008) showed that ABA can activate PsMPK2. Furthermore, the activation profile of PsMPK2 is similar to that described above for AtMPK1 and AtMPK2. JA and H_2_O_2_ also cause an increase in activity of this kinase, which in turn suggests that MAPKs may have the same functions across species in this context (Ortiz-Masia et al., 2008). In wild tobacco, *Nicotiana attenuata*, NaMPK4 plays a critical role in ABA-induced stomatal closure responses. *NaMPK4*-silenced plants (*irNaMPK4*) are impaired in their response to ABA- and H_2_O_2_-mediated stomatal closure. NPK4 is also involved in defense against aphids, invading pathogenic bacteria and *Alternaria alternata* (tobacco pathotype) (Slavov et al., 2004; Hettenhausen et al., 2012; Sun et al., 2014; Guo et al., 2017).

In cotton (*Gossypium hirsutum*), GhMPK17 expression is upregulated by ABA and also by NaCl. Notably, overexpression of GhMPK17 in *A. thaliana* results in increased tolerance to salt and osmotic stresses, as well as in changes in H_2_O_2_ levels and in the expression of stress-related genes (Zhang et al., 2014c). In recent work by Liu et al. (2016), a novel cotton *MAPKKK* gene, *GhMAPKKK49*, was isolated and shown to be significantly induced by exogenous treatment with ABA or H_2_O_2_. As GhMAPKKK49 also interacts with GhMKK4 and GhMKK9, it is tempting to hypothesize that a GhMAPKKK49–GhMKK4 or GhMAPKKK49–MKK9 cascade participates in ABA- and H_2_O_2_-mediated responses to abiotic stress (**Figure 6**). Recently, experimental work by Wang et al. (2016) showed that GhMKK3 plays an important role in drought tolerance by controlling the rate of water loss. Overexpression of *GhMKK3* in *N. benthamiana* results in more efficient ABA-induced stomatal closure and a decrease in the number of stomata (**Figure 5**). Intriguingly, both GhMKK3 and GhPIP1 interact with GhMPK7 to form a functional ABA- and drought-activated MAPK module. In support of this result, previous studies in Arabidopsis demonstrated that group C MAPKs, including AtMPK7, are activated by ABA in an MKK3-dependent manner (Danquah et al., 2015).

In *Brassica napus*, MKK1 appears to be involved in ABA signaling (**Supplementary Table 3**). Interestingly, overexpression of *BnMKK1* in transgenic tobacco plants causes rapid water loss, resulting in increased sensitivity to drought stress (Yu et al., 2014).

## Concluding Remarks and Future Prospects

In this review, we provide a comprehensive picture of our current understanding of the function of MAPKs and their interaction networks in plants. MAPK cascades are responsible for protein phosphorylation and signal transduction events associated with plant hormone signaling and therefore they play an essential role in the regulation of development, senescence, stress signaling and acclimation. Many cases of MAPK involvement in AUX, ABA, JA, SA, ET, and BR signaling have been identified and these demonstrate the complex structure, extensive crosstalk and dynamics of the signaling network. For example, analysis of MAPK function in ABA and ET signaling highlights MAPK regulation of target protein stability and the control of MAPK pathways by UPS degradation. However, despite the impressive current knowledge of MAPK cascades, their participation in BR and AUX signaling remains relatively unexplored. The very limited amount of data available on crosstalk between MAPK and GA signaling pathways (Huttly and Phillips, 1995; Marcote and Carbonell, 2000; Li Y. et al., 2014; Lu et al., 2015) highlights significant opportunities for further study in this area. In oat Aspk9 (also known as AsMAP1) seems to be negatively regulated by GA (Huttly and Phillips, 1995). The expression of the *PsMAPK3* gene is induced by GA in unpollinated pea ovary after fruit set (Marcote and Carbonell, 2000). Overexpression of GhMKK4 (homolog of AtMKK4 and AtMKK5) from *G. hirsutum* in transgenic *N. benthamiana* increases sensitivity of the plant to GA (as well as ABA) and significantly reduces GA levels after infection with *R. solanacearum* (Li Y. et al., 2014). In Chinese cabbage, BraMAPK17-2 and BraMAPK19-1 are upregulated by GA_3_, whereas transcript levels of most BraMAPKs, such as BraMAPK3, BraMAPK10-2/3, BraMAPK10-4/5, BraMAPK5, BraMAPK13, BraMAPK1. BraMAPK7-1, BraMAPK7-2, BraMAPK8-1 and BraMAPK16-2 are significantly lower after treatment with GA_3_ (Lu et al., 2015). Hypocotyl elongation in Arabidpopsis *mkk3-1* knockout seedlings is hypersensitive to exogenous GA_3_, and is proportional to GA_3_ concentration. However, no significant activation of MAPK cascade kinases has been observed in the Arabidopsis protoplast system after treatment with exogenous GA_3_, indicating that crosstalk may occur between AtMKK3 and GA in Arabidopsis, but is probably mediated through common downstream targets (Lee, 2015).

The molecular mechanisms that regulate MAPK assembly, activity (both activation and inactivation) and substrate binding require further elucidation. Nevertheless, several distinctive features of the mode of action of MAPKs have already been identified. The specificity of the MAPK module is achieved by coordinated expression of its components, by protein complex assembly and by subcellular localization (Danquah et al., 2014; Mitula et al., 2015; Bigeard and Hirt, 2018). In general, MAPK, MAPKK and MAPKKKs are localized in either nucleus or cytoplasm or both compartments. Localization in other cellular compartments is also evident. For example, MEKK1, besides being present in the nucleus and cytoplasm, can also be observed at the plasma membrane and in endosomes (Yang et al., 2010; Bigeard and Hirt, 2018). In addition, MAPK localization can be associated with its enzymatic activity. The active form of MAPKKK18 is localized in the nucleus, while the kinase-inactive isoform is found in the cytoplasm (Mitula et al., 2015). This suggests that not only MAPKKK18 activity but also the concentration of the protein is tightly controlled within the target compartment.

The activation of a typical MAPK module is rapid but transient. Subsequently, MAPK inactivation is achieved via dephosphorylation by dedicated protein phosphatases that function as part of a negative feedback loop to control the hormonal response. For example, MPK1 protein phosphatase regulates MPK3, MPK4 and MPK6 (Ulm et al., 2002; Bartels et al., 2009). Another MAPK phosphatase, MKP2, interacts with and controls the activity of MPK3 and MPK6 (Lumbreras et al., 2010). Furthermore, following ABA treatment, MPK3 and MPK6 kinase activities are inhibited by AP2C1 and PP2C5 phosphatases (Brock et al., 2010). Finally, MPK6 and MAPKKK18 are also regulated by the ABI1 protein phosphatase, a negative regulator of ABA signaling (Mitula et al., 2015). Besides protein phosphatases, activated MAPKs can be also controlled by the UPS dependent proteolytic pathway. For example ABA-regulated MAPKKK18 is regulated in this way (Mitula et al., 2015), demonstrating that MAPKKK18 downregulation by UPS is a significant factor in determining the nature of the MAPK signal output.

A consistent feature of MAPK function in hormone signaling is the existence of central MAPK-dependent hubs allowing extensive crosstalk between hormonal pathways, which leads to precisely regulated cellular functions. In principle, these MAPKs may use more sophisticated mechanisms to diversify signal outputs determined by different stimuli, such as tissue distribution and the formation of acontext-specific signalosome. The most prominent examples are AtMPK3/4/6. MPK3/6 are partially redundant in their activities and are crucial for coordinated response in scope of JA, SA, BR and ABA signaling pathway (**Figures 2**, **5**, **6**). AtMPK4 regulates crosstalk between the JA/ET/SA and AUX responses (**Figure 2**). All three kinases can autophosphorylate (Huang et al., 2000; Pecher et al., 2014) suggesting that they may be regulated by alternative mechanisms or potentially can even escape from the canonical model of MAPK activation. Another example is the AtMKK4/5-MPK6 module which precisely regulates ET and BR signaling and the corresponding responses (**Figures 3**, **4**).

The very complexity of MAPK cascades means that it is often difficult to define them in detail and to assign them a specific role in a particular biological process. Thus, to date, no MAPK cascade, together with its downstream substrates, has been defined in its entirety in any plant system. We also need a better characterization of the functional diversity and redundancy of MAPK complexes. MAPK cascades share many of their components, but nevertheless are still able to deliver hormonal signals to the cell interior precisely and specifically. Importantly, we also need to understand the consequences of phosphorylation by MAPKs for the function, localization or stability of their protein targets. Therefore, many questions remain, some of which are listed below:

• Which cellular elements function as molecular switches to support precise crosstalk and interaction outcomes between MAPK cascades? How do plants discriminate between hormone signaling pathways? How do MAPK cascades maintain specificity?
• What governs MAPK distribution within the cell? What post-transcriptional and translational mechanisms are employed to regulate this distribution?
• Which signaling systems are responsible for MAPK inactivation? How do these work? Which ligands target MAPK pathways to regulate their activity?
• What is the relative importance of different MAPK pathways in hormonal responses?

We believe that the answers to these questions will provide exciting discoveries and establish further the crucial role of MAPKs in plants. MAPK cascades, like other signaling networks, display a wide range of regulatory properties and the extension of MAPK research to all economically important crops is particularly relevant for ensuring sustainable food production globally.

## Author Contributions

PJ and AL designed the paper. All the authors wrote and revised the review. PJ, MT-Z, AC, and MM prepared the figures. PJ and MT-Z prepared the **Supplemental Figures** and **Supplementary Tables**.

## Conflict of Interest Statement

The authors declare that the research was conducted in the absence of any commercial or financial relationships that could be construed as a potential conflict of interest.
