# Supplementary figures and images for: Mitogen-Activated Protein Kinase Cascades in Plant Hormone Signaling
Source: Front Plant Sci. 2018 Oct 8;9:1387. doi: 10.3389/fpls.2018.01387 (PMC6187979; doi:10.3389/fpls.2018.01387)

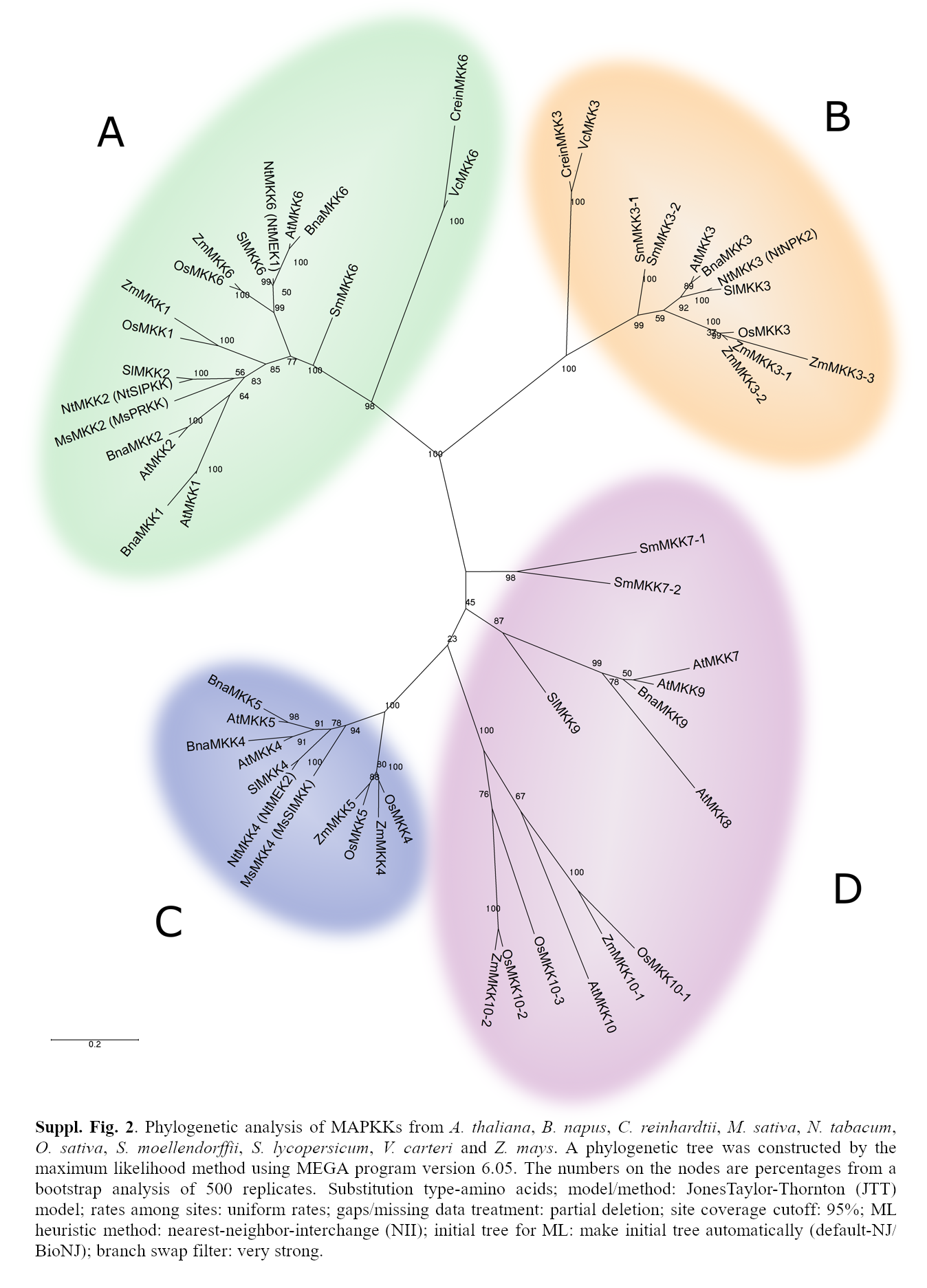

Supplement: Supplementary file 2 [file Image_2.tif]

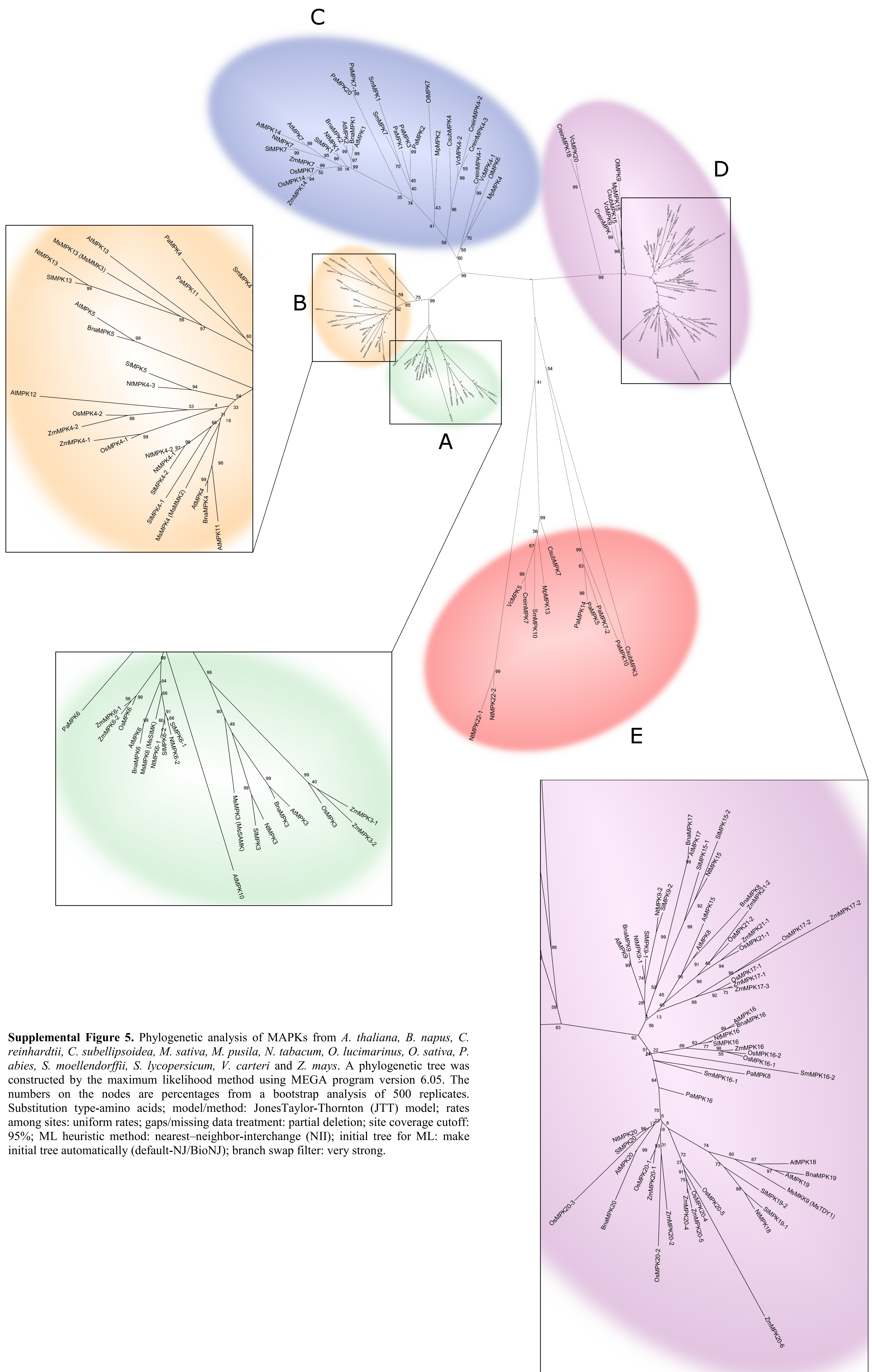

Supplement: Supplementary file 5 [file Image_5.pdf]
