# Supplementary material for: Mitogen-Activated Protein Kinase Cascades in Plant Hormone Signaling
Source: Front Plant Sci. 2018 Oct 8;9:1387. doi: 10.3389/fpls.2018.01387 (PMC6187979; doi:10.3389/fpls.2018.01387)
Supplement: Supplementary file 6 [file Table_1.pdf]

Supplemental Table 1. Nomenclature of plant MAPKKs divided by various groups based on sequence homology with *Arabidopsis* MAPKKs

Group	Gene name	ID / GenBank Acc No.	Polypeptide length (aa)	Other names	References
		Arabidopsis thaliana			
**A**	**AtMKK1**	At4g26070	354	AtMEK1	Hadiarto et al., 2006
	**AtMKK2**	At4g29810	363		Teige et al., 2004
	**AtMKK6**	At5g56580	356	AtANQ1	Kosetsu et al. 2010
**B**	**AtMKK3**	At5g40440	520		Lee et al., 2008a
**C**	**AtMKK4**	At1g51660	366		Le et al., 2014; Lau and Bergmann 2012; Kim et al., 2012a
	**AtMKK5**	At3g21220	348	AtMEK5, AtMAP2Kalpha	Liu et al., 2008; Huang et al. 2000
**D**	**AtMKK7**	At1g18350	307	AtBUD1	Dai et al., 2006
	**AtMKK8**	At3g06230	293		
	**AtMKK9**	At1g73500	310		Lee et al., 2008a
	**AtMKK10**	At1g32320	305		Lee et al., 2008a
		Brassica napus			
**A**	**BnaMKK1**	JQ708028	355		Liang et al. 2013
	**BnaMKK2**	JQ708029	364		Liang et al. 2013
	**BnaMKK6**	JQ708032	356		Liang et al. 2013
**B**	**BnaMKK3**	JQ708030	518		Liang et al. 2013
**C**	**BnaMKK4**	JQ708031	356		Liang et al. 2013
	**BnaMKK5**	KC246595	332		Liang et al. 2013
**D**	**BnaMKK9**	JQ708033	306		Liang et al. 2013
		Chlamydomonas reinhardtii			
**A**	**CreinMKK6**	XP_001693665	249		
**B**	**CreinMKK3**	XP_001696437	452		
		Medicago sativa			
**A**	**MsMKK2**	AJ293275	356	MsPRKK	Cardinale et al., 2002
**C**	**MsMKK4**	AJ293274	368	MsSIMKK	Cardinale et al., 2002
		Nicotiana tabacum			
**A**	**NtMKK2**	AF165186	357	NtSIPKK	Gomi et al., 2005
	**NtMKK6**	AJ302651	354	NtMEK1, NtNQK1	Soyano et al., 2003
**B**	**NtMKK3**	D31964	518	NtNPK2	Gomi et al., 2005
**C**	**NtMKK4**	AF325168	372	NtMEK2	Gomi et al., 2005
		Oryza sativa			
**A**	**OsMKK1**	Os06g05520	352	OsMEK2	Singh et al., 2012
	**OsMKK6**	Os01g32660	355	OsMEK1	Singh et al., 2012
**B**	**OsMKK3**	Os06g27890	320	OsMEK8a, OsMEK8b[9]	Singh et al., 2012
**C**	**OsMKK4**	Os02g54600	369	OsMEK6	Singh et al., 2012
	**OsMKK5**	Os06g09180	342	OsMEK7a, OsMEK7b	Singh et al., 2012
**D**	**OsMKK10-1**	Os02g46760	340	OsMEK4	Singh et al., 2012
	**OsMKK10-2**	Os03g12390	339	OsMEK3	Singh et al., 2012
	**OsMKK10-3**	Os03g50550	345		

Selaginella moellendorffii					
**A**	**SmMKK6**	272204	346		
**B**	**SmMKK3-1**	74697	509		
	**SmMKK3-2**	168690	509		
**D**	**SmMKK7-1**	90224	333		
	**SmMKK7-2**	186197	353		
Solanum lycopersicum					
**A**	**SlMKK2**	Solyc12g009020	357	LeMEK1, SlMKK1	Cardinale et al., 2002; Li et al., 2014a
	**SlMKK6**	Solyc03g119490	354	SlMKK3	Li et al., 2014a
**B**	**SlMKK3**	Solyc03g019850	515	SlMKK5	Li et al., 2014a
**C**	**SlMKK4**	Solyc03g123800	359	SlMKK2	Li et al., 2014a
**D**	**SlMKK9**	Solyc03g097920	335	SlMKK4	Li et al., 2014a
Volvox carteri					
**A**	**VcMKK6**	73333	415		
**B**	**VcMKK3**	99491	327		
Zea Mays					
**A**	**ZmMKK1**	GRMZM2G400470_T03	350		
	**ZmMKK6**	GRMZM2G167856_T01	355	ZmMEK1	Kong et al., 2013
**B**	**ZmMKK3-1**	JN972438	523	ZmMKK3	Kong et al., 2013
	**ZmMKK3-2**	GRMZM2G004468_T05	561		
	**ZmMKK3-3**	GRMZM2G367411_T01	299		
**C**	**ZmMKK4**	GU942956	357		
	**ZmMKK5**	GRMZM5G834697_T01	347	ZmMAPKK1	Kong et al., 2013
**D**	**ZmMKK10-1**	GRMZM2G130213_T01	375		
	**ZmMKK10-2**	GRMZM2G344388_T01	401		
