# Supplementary material for: Mitogen-Activated Protein Kinase Cascades in Plant Hormone Signaling
Source: Front Plant Sci. 2018 Oct 8;9:1387. doi: 10.3389/fpls.2018.01387 (PMC6187979; doi:10.3389/fpls.2018.01387)
Supplement: Supplementary file 7 [file Table_2.pdf]

Supplemental Table 2 Nomenclature of plant MAPKs divided on groups based on sequence homology with *Arabidopsis* MAPKs

Motif	Group	Gene name	ID / GenBank Acc No.	Polypeptide length (aa)	Other names	References
			Arabidopsis thaliana			
T-E-Y	MAPK group A	**AtMPK3**	At3g45640	370		Droillard et al., 2002
		**AtMPK6**	At2g43790	395		Droillard et al., 2002
		**AtMPK10**	At3g59790	393		Lee et al., 2008a
	MAPK group B	**AtMPK4**	At4g01370	376		Teige et al., 2004; Kong et al., 2012; Rodriguez et al., 2010; Kosetsu et al., 2010
		**AtMPK5**	At4g11330	376		Teige et al., 2004; Lee et al., 2008a
		**AtMPK11**	At1g01560	369		Lee et al., 2008a
		**AtMPK12**	At2g46070	372		Meszaros et al., 2006; Lee et al., 2009
		**AtMPK13**	At1g07880	363		Teige et al., 2004; Lee et al., 2008a
	MAPK group C	**AtMPK1**	At1g10210	370		Lee et al., 2008a; Doczi et al., 2012
		**AtMPK2**	At1g59580	376		Lee et al., 2008a; Doczi et al., 2012
		**AtMPK7**	At2g18170	368		Lee et al., 2008a; Doczi et al., 2012
		**AtMPK14**	At4g36450	361		Lee et al., 2008a; Doczi et al., 2012
T-D-Y	MAPK group D	**AtMPK8**	At1g18150	589		Doczi et al., 2012
		**AtMPK9**	At3g18040	510		Doczi et al., 2012
		**AtMPK15**	At1g73670	576		Doczi et al., 2012
		**AtMPK16**	At5g19010	567		Doczi et al., 2012
		**AtMPK17**	At2g01450	486		Lee et al., 2008a
		**AtMPK18**	At1g53510	615		Wang et al., 2011; Doczi et al., 2012
		**AtMPK19**	At3g14720	598		Doczi et al., 2012
		**AtMPK20**	At2g42880	606		Wang et al., 2011; Doczi et al., 2012; Lee et al., 2008a
			Brassica napus			
T-E-Y	MAPK group A	**BnaMPK3**	JQ708040	370		Liang et al. 2013
		**BnaMPK6**	JQ708043	395		Liang et al. 2013
	MAPK group B	**BnaMPK4**	JQ708041	373		Liang et al. 2013
		**BnaMPK5**	JQ708042	373		Liang et al. 2013
	MAPK group C	**BnaMPK1**	JQ708034	369		Liang et al. 2013
		**BnaMPK2**	JQ708038	370		Liang et al. 2013
T-D-Y	MAPK group D	**BnaMPK8**	JQ708044	581		Liang et al. 2013
		**BnaMPK9**	JQ708045	501		Liang et al. 2013
		**BnaMPK16**	JQ708035	558		Liang et al. 2013
		**BnaMPK17**	JQ708036	487		Liang et al. 2013
		**BnaMPK19**	JQ708037	599		Liang et al. 2013
		**BnaMPK20**	JQ708039	611		Liang et al. 2013
			Chlamydomonas reinhardtii			
T-E-Y	MAPK group C	**CreinMPK4-1**	Cre01.g010000.t1.2	389		Mohanta et al., 2015
		**CreinMPK4-2**	Cre12.g508900.t1.2	375		Mohanta et al., 2015
		**CreinMPK4-3**	Cre12.g509000.t1.3	353		Mohanta et al., 2015
T-D-Y	MAPK group D	**CreinMPK15**	Cre08.g385050.t1.3	740		Mohanta et al., 2015

		**CreinMPK18**	g18025.t1	765		Mohanta et al., 2015
	MAPK group E	**CreinMPK7**	Cre13.g607300.t1.2	433		Mohanta et al., 2015
Coccomyxa subellipsoidea						
**T-E-Y**	MAPK group C	**CsubMPK4**	11831	377		Mohanta et al., 2015
**T-D-Y**	MAPK group D	**CsubMPK15**	54284	537		Mohanta et al., 2015
**T-R-M**	MAPK group E	**CsubMPK3**	14112	359		Mohanta et al., 2015
**T-D-Y**	MAPK group E	**CsubMPK7**	47764	391		Mohanta et al., 2015
Medicago sativa						
**T-E-Y**	MAPK group A	**MsMPK3**	X82270	371	MsSAMK, MsMMK4	Cardinale et al., 2002; Daxberger et al., 2007
**T-E-Y**	MAPK group A	**MsMPK6**	X66469	387	MsSIMK, MsK7, MsMMK1	Cardinale et al., 2002; Jonak et al., 1993; Daxberger et al., 2007
**T-E-Y**	MAPK group B	**MsMPK4**	X82268	371	MsMMK2	Cardinale et al., 2002
**T-E-Y**	MAPK group B	**MsMPK13**	AJ224336	374	MsMMK3	Cardinale et al., 2002
**T-D-Y**	MAPK group D	**MsMPK19**	AF129087	608	MsTDY1	MAPK Group 2002
***Micromonas pusila* CCMP1545**						
**T-E-Y**	MAPK group C	**MpMPK2**	203785	374		Mohanta et al., 2015
**T-E-Y**	MAPK group C	**MpMPK4**	27380	426		Mohanta et al., 2015
**T-D-Y**	MAPK group D	**MpMPK15**	44271	362		Mohanta et al., 2015
**T-D-Y**	MAPK group E	**MpMPK13**	14294	380		Mohanta et al., 2015
Nicotiana tabacum						
**T-E-Y**	MAPK group A	**NtMPK3**	AB052964	375	NtWIPK, NtMPK5	MAPK Group 2002; Zhang et al., 2013
**T-E-Y**	MAPK group A	**NtMPK6-1**	AF165186	357	NtSIPK, NtMPK6	MAPK Group 2002; Zhang et al., 2013
**T-E-Y**	MAPK group A	**NtMPK6-2**	X83880	393	NtNTF4, NtMPK7	MAPK Group 2002; Zhang et al., 2013
**T-E-Y**	MAPK group B	**NtMPK4-1**	AB212070	373	NtMPK4, NtMPK1	Gomi et al., 2005; Zhang et al., 2013
**M-E-Y**	MAPK group B	**NtMPK4-2**	JX076820	374	NtMPK2	Zhang et al., 2013
**T-E-Y**	MAPK group B	**NtMPK4-3**	JX076821	380	NtMPK3	Zhang et al., 2013
**T-E-Y**	MAPK group B	**NtMPK13**	AB055515	371	NtNRK1, NtNTF6, NtMPK4	MAPK Group 2002; Zhang et al., 2013
**T-E-Y**	MAPK group C	**NtMPK1**	X69971	372	NtMPK14, NtNTF3	Berberich et al., 1999; MAPK Group 2002
**T-E-Y**	MAPK group C	**NtMPK7**	JX076817	370	NtMPK15	Zhang et al., 2013
**T-D-Y**	MAPK group D	**NtMPK9-1**	JX076815	594	NtMPK12	Zhang et al., 2013
**T-D-Y**	MAPK group D	**NtMPK9-2**	JX076816	590	NtMPK13	Zhang et al., 2013
**T-D-Y**	MAPK group D	**NtMPK15**	JX076814	509	NtMPK11	Zhang et al., 2013
**T-D-Y**	MAPK group D	**NtMPK16**	JX076813	566	NtMPK10	Zhang et al., 2013
**T-D-Y**	MAPK group D	**NtMPK18**	JX076823	373	NtMPK9	Zhang et al., 2013
**T-D-Y**	MAPK group D	**NtMPK20**	JX076822	610	NtMPK8	Zhang et al., 2013
**T-E-Y**	MAPK group E	**NtMPK22-1**	JX076818	425	NtMPK16	Zhang et al., 2013
**T-E-Y**	MAPK group E	**NtMPK22-2**	JX076819	470	NtMPK17	Zhang et al., 2013
Ostreococcus lucimarinus						
**T-E-Y**	MAPK group C	**OlMPK6**	C_Chr_9000126	405		Mohanta et al., 2015
**T-S-Y**	MAPK group C	**OlMPK7**	e_gwEuk.9.247	449		Mohanta et al., 2015
**T-D-Y**	MAPK group D	**OlMPK9**	gwEuk.8.109.1	387		Mohanta et al., 2015
Oryza sativa						

T-E-Y	MAPK group A	**OsMPK3**	Os03g17700	369	OsMPK5, OsMAP1, OsMAPK2, OsMSRMK2, OsBIMK1	Reyna and Yang, 2006; Wen et al., 2002; Huang et al., 2002; Agrawal et al., 2002; Song and Goodman, 2002
		**OsMPK6**	Os06g06090	398	OsMPK1, OsMAPK6, OsSIPK	Reyna and Yang, 2006; Lieberherr et al., 2005; Lee et al., 2008b
	MAPK group B	**OsMPK4-1**	Os10g38950	376	OsMPK6	Reyna and Yang, 2006
M-E-Y		**OsMPK4-2**	Os08g06060	394	OsMPK2	Reyna and Yang, 2006
T-E-Y	MAPK group C	**OsMPK7**	Os06g48590	369	OsMPK4, OsMAPK4, OsMAPkinase2, OsMSRMK3, OsBIMK1, OsMPK3	Reyna and Yang, 2006; Jonak et al., 1993; Song and Goodman, 2002; Hamel et al., 2006
		**OsMPK14**	Os02g05480	370	OsMPK3, OsMAPK3, OsMAP3	Reyna and Yang, 2006; Jonak et al., 1993; Cardinale et al., 2002
T-D-Y	MAPK group D	**OsMPK16-1**	Os11g17080	498	OsMPK15	Reyna and Yang, 2006
		**OsMPK16-2**	Os05g05160	542	OsMPK14	Reyna and Yang, 2006
		**OsMPK17-1**	Os06g49430	580	OsMPK12, OsBWMK1	Reyna and Yang, 2006; Cheong et al., 2003
		**OsMPK17-2**	Os02g04230	506	OsMPK13, OsBIMK2, OsRMAPK2	Reyna and Yang, 2006; Song et al., 2006; Hamel et al., 2006
		**OsMPK20-1**	Os01g43910	611	OsMPK9, OsMPK10	Reyna and Yang, 2006
-		**OsMPK20-2**	Os05g50560	349		
T-D-Y		**OsMPK20-3**	Os06g26340	570	OsMPK11	Reyna and Yang, 2006
		**OsMPK20-4**	Os01g47530	569	OsMPK8, OsWJUMK1, OsMPKG1	Reyna and Yang, 2006; Hamel et al., 2006
		**OsMPK20-5**	Os05g49140	592	OsMPK7	Reyna and Yang, 2006
		**OsMPK21-1**	Os05g50120	582	OsMPK17	Reyna and Yang, 2006
		**OsMPK21-2**	Os01g45620	501	OsMPK16, OsMPKG2	Reyna and Yang, 2006; Hamel et al., 2006
Picea abies						
T-E-Y	MAPK group A	**PaMPK6**	MA_10437020g0010	469		Mohanta et al., 2015
	MAPK group B	**PaMPK4**	MA_10437018g0010	423		Mohanta et al., 2015
		**PaMPK11**	MA_10432928g0010	290		Mohanta et al., 2015
	MAPK group C	**PaMPK1**	MA_10428223g0010	368		Mohanta et al., 2015
		**PaMPK2**	MA_9261976g0010	179		Mohanta et al., 2015
		**PaMPK3**	MA_160202g0010	179		Mohanta et al., 2015
-		**PaMPK7-1**	MA_8212817g0010	180		Mohanta et al., 2015
		**PaMPK20**	MA_8212817g0010	180		Mohanta et al., 2015
T-D-Y	MAPK group D	**PaMPK8**	MA_10427605g0010	216		Mohanta et al., 2015
		**PaMPK16**	MA_91728g0010	749		Mohanta et al., 2015
T-E-M	MAPK group E	**PaMPK5**	MA_42176g0010	404		Mohanta et al., 2015
		**PaMPK7-2**	MA_60199g0010	145		Mohanta et al., 2015
T-Q-M		**PaMPK10**	MA_117156g0010	364		Mohanta et al., 2015
T-E-M		**PaMPK14**	MA_42176g0010	404		Mohanta et al., 2015
Selaginella moellendorffii						
T-E-Y	MAPK group B	**SmMPK4**	105143	374		Mohanta et al., 2015
	MAPK group C	**SmMPK1**	443152	374		Mohanta et al., 2015
		**SmMPK7**	75282	370		Mohanta et al., 2015

**T-D-Y**	MAPK group D	**SmMPK16-1**	74687	474		Mohanta et al., 2015
		**SmMPK16-2**	97841	408		Mohanta et al., 2015
	MAPK group E	**SmMPK10**	82767	380		Mohanta et al., 2015
		Solanum lycopersicum				
**T-E-Y**	MAPK group A	**SlMPK3**	Solyc06g005170	373	SlMAPK3, LeMPK3	Kong et al., 2012; Kandoth et. al., 2007
		**SlMPK6-1**	Solyc08g014420	394	SlMAPK2, LeMPK2, SlMPK2	Kong et al., 2012; Kandoth et. al., 2007; Li et al., 2014a
		**SlMPK6-2**	Solyc12g019460	396	SlMAPK1, LeMPK1, SlMPK1	Kong et al., 2012; Kandoth et. al., 2007; Li et al., 2014a
**M-E-Y**	MAPK group B	**SlMPK4-1**	Solyc05g049970	376	SlMAPK6	Kong et al., 2012
**T-E-Y**		**SlMPK4-2**	Solyc01g094960	373	SlMAPK5	Kong et al., 2012
		**SlMPK5**	Solyc08g081490	379	SlMAPK7	Kong et al., 2012
		**SlMPK13**	Solyc11g072630	372	SlMAPK4, SlMPK4	Kong et al., 2012; Li et al., 2014a;
	MAPK group C	**SlMPK1**	Solyc04g080730	372	SlMAPK9	Kong et al., 2012
		**SlMPK7**	Solyc02g084870	370	SlMAPK8	Kong et al., 2012
**T-D-Y**	MAPK group D	**SlMPK9-1**	Solyc06g068990	601		
		**SlMPK9-2**	Solyc12g040680	575	SlMAPK16	Kong et al., 2012
		**SlMPK15-1**	Solyc04g007710	512	SlMAPK14	Kong et al., 2012
		**SlMPK15-2**	Solyc05g008020	513	SlMAPK15	Kong et al., 2012
		**SlMPK16**	Solyc01g080240	565	SlMAPK13	Kong et al., 2012
		**SlMPK19-1**	Solyc10g007500	593	SlMAPK10	Kong et al., 2012
		**SlMPK19-2**	Solyc07g062080	600	SlMAPK11	Kong et al., 2012
		**SlMPK20**	Solyc07g056350	621	SlMAPK12	Kong et al., 2012
		Volvox carteri				
**T-E-Y**	MAPK group C	**VcMPK4-1**	Vocar20007163m	397		Mohanta et al., 2015
		**VcMPK4-2**	Vocar20000906m	381		Mohanta et al., 2015
**T-D-Y**	MAPK group D	**VcMPK9**	Vocar20004345m	466		Mohanta et al., 2015
		**VcMPK20**	Vocar20011355m.g	644		Mohanta et al., 2015
	MAPK group E	**VcMPK5**	Vocar20007415m	430		Mohanta et al., 2015
		Zea mays				
**T-E-Y**	MAPK group A	**ZmMPK3-1**	GRMZM2G053987	321	ZmMPK4; ZmMPK5	Sun et al., 2015; Berberich et al., 1999
		**ZmMPK3-2**	GRMZM2G017792	376	ZmMPK3	Sun et al., 2015
		**ZmMPK6-1**	GRMZM2G002100	398	ZmMPK7	Sun et al., 2015
		**ZmMPK6-2**	GRMZM2G020216	398	ZmMPK5	Sun et al., 2015
	MAPK group B	**ZmMPK4-1**	GRMZM2G127141	372	ZmSIMK, ZmMPK4	Sun et al., 2015; Liu et al., 2013
**M-E-Y**		**ZmMPK4-2**	GRMZM2G123886	391	ZmMPK1, ZmMPK12	Sun et al., 2015; Liu et al., 2013
**T-E-Y**	MAPK group C	**ZmMPK7**	GRMZM2G048455	369	ZmMPK8, ZmMPK1	Sun et al., 2015; Liu et al., 2013
		**ZmMPK14**	GRMZM2G062914	370	ZmMPK2	Sun et al., 2015
**T-D-Y**	MAPK group D	**ZmMPK16**	GRMZM2G089484	557	ZmMPK6	Sun et al., 2015;
		**ZmMPK17-1**	GRMZM2G306028	499	ZmMPK10, ZmMPK17-2	Sun et al., 2015; Liu et al., 2013
		**ZmMPK17-2**	GRMZM2G374088	491	ZmMPK17, ZmMPK17-1	Sun et al., 2015; Liu et al., 2013
		**ZmMPK17-3**	GRMZM2G135904	289	ZmMPK9	Sun et al., 2015
		**ZmMPK20-1**	GRMZM2G131334	483	ZmMPK14, ZmMPK20-2	Sun et al., 2015; Liu et al., 2013
		**ZmMPK20-2**	GRMZM2G163861	602	ZmMPK13, ZmMPK20-1	Sun et al., 2015; Liu et al., 2013
		**ZmMPK20-4**	GRMZM2G122335	588	ZmMPK19, ZmMPK18-1	Sun et al., 2015; Liu et al., 2013
		**ZmMPK20-5**	GRMZM2G007848	589	ZmMPK18, ZmMPK18-2	Sun et al., 2015; Liu et al., 2013

		**ZmMPK20-6**	GRMZM2G034052	579	ZmMPK16, ZmMPK19	Sun et al., 2015; Liu et al., 2013
		**ZmMPK21-1**	GRMZM2G062761	601	ZmMPK12, ZmMPK15	Sun et al., 2015; Liu et al., 2013
		**ZmMPK21-2**	GRMZM2G375975	489	ZmMPK11, ZmMPK8	Sun et al., 2015; Liu et al., 2013
