# Supplementary material for: Mitogen-Activated Protein Kinase Cascades in Plant Hormone Signaling
Source: Front Plant Sci. 2018 Oct 8;9:1387. doi: 10.3389/fpls.2018.01387 (PMC6187979; doi:10.3389/fpls.2018.01387)
Supplement: Supplementary file 8 [file Table_3.pdf]

**Supplemental Table 3.** Data on the ABA- regulated gene coding MAP kinase.

| | Annotation | ID / GenBank Acc No. | Homologus proteins | Other factors leading to the up-($^{U}$) and down-regulation ($^{D}$) of MAPK genes | Known MAP kinase interacting partner(s) | References |
|---|---|---|---|---|---|---|
| **UP-REGULATED GENES** **_Arachis hypogaea_ (peanut)** | | | | | | |
| MAPK (A group) | ***AhMPK3*** | EU182580 | GmMPK1, MsMMK4, CsTIPK, PtMPK3-1, AtMPK3, NtWIPK, LeMPK3 | wounding stress$^{U}$ , $H_2O_2$$^{U}$ , SA$^{U}$ ,MeJA $^{U}$ | - | Kumar et al., 2009 |
| ***Brassica napus (canola)*** | | | | | | |
| MAPKK (A group) | ***BnaMKK1*** | HQ916282 | AtMKK1,*OsMKK1* | SA$^{U}$ , *S. sclerotiorum* $^{U}$ (pathogenic fungus) | BnaMPK6 | Liang et al.,2013, Sun et al., 2014 Yu et al., 2014 |
| MAPK (B group) | ***BnOIPK*** | - | AtMPK4 | Chitosan$^{U}$, JA$^{U}$ , SA $^{U}$ | - | Yin et al., 2010 |
| ***Brassica rapa* (field mustard)** | | | | | | |
| MAPK (B group) | ***BraMAPK5*** | Bra035233* | AtMAPK5 | waterlogging$^{U}$ ,NaCl$^{U}$ , cold$^{U}$ and wound$^{D}$ stress , 6-BA$^{U}$ , BR$^{U}$ , NAA$^{U}$, IAA$^{U}$ | **-** | Lu et al., 2015 |
| MAPK (D group) | ***BraMAPK17-1*** | Bra026665* | AtMAPK17 | BR $^{U}$, heat$^{U}$ and cold$^{U}$ stress, | **-** | Lu et al., 2015 |
| MAPK (D group) | ***BraMAPK17-2*** | Bra024886* | AtMAPK17 | heat$^{U}$ , NaCl$^{U}$ , PEG$^{U}$ , waterlogging$^{U}$ , wound$^{U}$ and cold$^{D}$ stress**,** 6-BA$^{U}$ , BR$^{U}$,GA3$^{U}$ , NAA$^{U}$ , IAA$^{U}$ | **-** | Lu et al., 2015 |
| MAPK (D group) | ***BraMAPK18-1*** | Bra038128* | AtMAPK18 | NaCl$^{U}$, PEG$^{U}$, cold$^{U}$, waterlogging stress$^{U}$ | - | Lu et al., 2015 |
| MAPK (D group) | ***BraMAPK19-1*** | Bra027317* | *AtMAPK19* | NaCl$^{U}$ , PEG$^{U}$ cold$^{U}$, waterlogging$^{U}$, heat stress$^{U}$ | - | Lu et al., 2015 |
| ***Capsicum annuum* (pepper)** | | | | | | |
| MAPK (D group) | ***CaMPK9-1*** | CA12g10140 | AtMPK8, AtMPK15, AtMPK9 | NaCl$^{U}$, plant pathogenic bacterium (*Ralstonia solanacearum*)$^{U}$ | - | Liu et al., 2015 |
| MAPK (D group) | ***CaMPK17-1*** | CA05g08630 | AtMPK15 | plant pathogenic bacterium (*Ralstonia solanacearum*)$^{U}$ | **-** | Liu et al., 2015 |
| ***Chorispora bungeana*** | | | | | | |

| | | | | | | |
|---|---|---|---|---|---|---|
| MAPK (A group) | ***CbMAPK3*** | AY805424 | AtMPK3, NtWIPK, CaMPK1, PsMAPK3, MsMMK4, Petrosecinum crispum PARSLEY MAPK | NaCl[U], cold stress[U] | - | Zhang et al., 2006 |
| ***Curcuma longa L.*** **(turmeric)** | | | | | | |
| MAPK (A group) | ***ClMPK6*** | *KP782042* | AtMPK6, ZmMPK6, BnMPK6, OsMPK6 | MeJA[U] , SA[U], GA[U] | - | Nanda et al., 2016 |
| ***Gossypium hirsutum*** **(upland cotton*)*** | | | | | | |
| MAPKKK (Raf-like) | ***GhMAPKKK49*** | KP057883 | SlMAPKKK24, ZmMAPKKK57, AtRaf41, BnaRaf41 | drought[U] and wounding stress[U], $H_2O_2$[U], GA[U], MeJA[U], SA[U], 6-BA[U], NAA[U], ET[U] | GhMAPKK4, GhMAPKK9 | Liu et al., 2016 |
| MAPKK (A group) | ***GhMKK2_2*** | FJ966886.1 | AtMKK2 | SA[U],JA[U], $H_2O_2$[U], wounding stress[U] | GhMPK3, GhMPK6, GhMPK18, GhMPK12, GhMPK14 GhMPK19, GhMPK23, GhMPK27 | Zhang et al., 2016 |
| MAPKK (C group) | ***GhMKK4*** | **-** | AtMKK4 | SA[U],wounding stress[U] | GhMPK8, GhMPK16, GhMPK20 | Li et al., 2014, Zhang et al., 2016 |
| MAPKK (C group) | ***GhMKK5*** | HQ637469.1 | AtMKK5 | SA[U],$H_2O_2$[U], wounding stress[U] | GhMPK3, GhMPK6, GhMPK12, GhMPK13, GhMPK27 | Zhang et al., 2016 |
| MAPKK (A group) | ***GhMKK6*** | GU188977.1 | AtMKK6 | SA[U],JA[U], $H_2O_2$[U], PEG[U], drought[U] , heat[U] , wounding[U] , and cold stress[U] | GhMPK3, GhMPK6, GhMPK16 GhMPK18, | Zhang et al., 2016 |
| MAPKK (D group) | ***GhMKK7*** | - | AtMKK7 | SA[U],JA[U], $H_2O_2$[U], drought[U], heat[U] , wounding[U]and cold stress[U] | GhMPK8, GhMPK14, GhMPK16, GhMPK25 | Zhang et al., 2016 |
| MAPKK (D group) | ***GhMKK10_1*** | - | AtMKK10 | SA[U],JA[U], $H_2O_2$[U], cold[U] and wounding stress[U] | 17 of the 21 MAPK genes (no interaction: GhMPK5, GhMPK10, GhMPK14, GhMPK25) | Zhang et al., 2016 |
| MAPKK | ***GhMPK11*** | KP901089 | TcMPK4, AtMPK11 and AtMPK4, | $GA_3$[U],pathogens(*R. Solanacearum*, | - | Wang et al., 2016, |

| | | | | | | |
|---|---|---|---|---|---|---|
| (D group) | | | NaMPK4 | *R. solani*)$^{U}$ | | Zhang et al., 2016 |
| MAPKK<br>(D group) | ***GhMPK17*** | *KJ192192* | AtMPK17, ZmMPK17 , OsMPK17-2 | PEG$^{U}$ and cold stress$^{U}$ | - | Zhang et al., 2014b |
| ***Ipomoea batatas* (sweet potato)** | | | | | | |
| MAPK<br>(A group) | ***IbMPK3*** | *KT934378* | *NtWIPK 22, CaMPK1, PsMAPK3, AtMPK3, MsMMK4* | NaCl$^{U}$, $H_2O_2^{U}$,cold stress$^{U}$, SA$^{U}$,JA$^{U}$ | - | Kim et al., 2016 |
| MAPK<br>(A group) | ***IbMPK6*** | *KT934379* | *NtSIPK, CaMAPK2, MsMMK1, AtMPK6, PsMAPK* | salinity stress$^{U}$, $H_2O_2^{U}$, SA$^{U}$, JA$^{U}$ | - | Kim et al, 2016 |
| ***Malus domestica* (apple)** | | | | | | |
| MAPK<br>(A group) | ***MdMPK1*** | *GU139791.1* | PcMKK2, NtSIPKK, LeMEK1, AtMKK2 | - | MdMKK1 | Wang et al., 2010b |
| ***Musa acuminata L.* (banana)** | | | | | | |
| MAPK<br>(D group) | ***MaMAPK2*** | *KF582546* | AtMPK20 | ETH$^{U}$, MeJA$^{U}$, SA$^{U}$, pathogenic fungus( Fusarium Oxysporum f. Sp)$^{U}$ | - | Wang et al., 2015b |
| MAPK<br>(D group) | ***MaMAPK3*** | KF594427 | AtMPK9, AtMPK17, OsMPK12, OsMPK13 | ETH$^{U}$ MeJA$^{U}$, SA$^{U}$, pathogenic fungus( *Fusarium Oxysporum f. Sp*)$^{U}$ | - | Wang et al., 2015b |
| MAPK<br>(B group) | ***MaMAPK4*** | KF594427 | OsMPK2, AtMPK4, AtMPK11 | ABA$^{U}$, ETH$^{U}$, MeJA$^{U}$, SA$^{U}$ | - | Wang et al., 2015b |
| MAPK<br>(D group) | ***MaMAPK6*** | KF594427 | OsMPK11 | ABA$^{U}$, ETH$^{U}$, MeJA$^{U}$, SA$^{U}$, pathogenic fungus (F*usarium Oxysporum f. Sp*)$^{U}$ | - | Wang et al., 2015b |
| ***Manihot esculenta* (cassava)** | | | | | | |
| MAPK<br>(A group) | ***MeMAPK1*** | - | CsMPK6, MDMPK6-1, MDMPK6-2, SIMAPK1, SIMAPK2, AtMPK6, OsMPK6 | PEG$^{U}$, $H_2O_2^{D}$, NaCl$^{U}$, cold stress$^{D}$ | - | Yan et al., 2016 |
| MAPK<br>(B group) | ***MeMAPK10*** | - | MdMPK4-4, CsMPK4-1 | PEG$^{U}$, cold stress$^{D}$, NaCl$^{U}$ | - | Yan et al., 2016 |
| MAPK<br>(B group) | ***MeMAPK17*** | - | CsMPK4-2, MdMPK4-1, MdMPK4-2, SiMAPK5, SIMAPK6 | NaCl$^{U}$, $H_2O_2^{D}$, cold stress$^{D}$ | - | Yan et al., 2016 |
| ***Origanum onites L.* (Lamiaceae)** | | | | | | |
| MAPKK<br>(A group) | ***OoMAPKK1*** | *EF558371* | NtMEK1, LeMKK3, AtMKK6, OsMEK1, OsMAPKK6, ZmMEK1 | cold$^{U}$ and wounding stress$^{U}$ | - | Poyraz, 2013 |
| ***Oryza minuta*** | | | | | | |
| MAPKK | ***OmMKK1*** | *DQ989217* | *OsMKK1,* OsMKK6 AtMKK1, AtMKK2 , | MeJA$^{U}$, ETH$^{U}$, SA$^{U}$, MV$^{U}$ | - | You et al., 2007 |

| | | | | | | |
|---|---|---|---|---|---|---|
| (A group) | | | AtMKK6 | | | |
| ***Oryza sativa*** | | | | | | |
| MAPK<br>(A group) | ***OsMPK1* (*OsMPK6, OsMAPK6, OsSIPK*)** | AJ535841 | NtSIPK, AtMPK6, MsSIMK | JA$^{U}$, SA$^{U}$, ETH$^{U}$, CHX$^{U}$, JA/SA + CHX$^{U}$, cantharidin$^{U}$, okadaic acid$^{U}$, $H_2O_2^{U}$, chitosan$^{U}$, Nacl$^{U}$, cold stress$^{U}$ | OsMKK4 | Lee et al., 2008b,<br>Zhang et al., 2012b,<br>Yoo et al., 2014 |
| MAPK<br>(C group) | ***OsMPK4* (*OsMAPK4, OsMPK7, OsMAPkinase2, OsMPK2, OsMSRMK3, OsMPK3*)** | AJ512642 | OsMAPK3, AtMPK1 | wounding$^{U}$, JA$^{U}$, SA$^{U}$, ET$^{U}$, $H_2O_2^{U}$, protein phosphatase inhibitors$^{U}$, chitosan$^{U}$, high salt/sugar$^{U}$, heavy metals$^{U}$ | - | Agrawal et al., 2003a,<br>Reyna et al., 2006 |
| MAPK<br>(A group) | ***OsMPK3* (*OsMPK5, OsMAP1, OsMAPK2, OsMAPK5, OsMSRMK2, OsBIMK1*)** | AJ486975 | AtMPK3, ZmMAPK4, NtSIPK, NtWIPK, | $H_2O_2^{U}$, SA$^{U}$,BTH $^{U}$, dichloroisonicotinic acid $^{U}$, probenazole $^{U}$, JA and its methyl ester $^{U}$, plant pathogenic bacteria (*Pseudomonas syringae* pv. *Syringae*)$^{U}$, wounding $^{U}$ | OsMKK6 | Agrawal et al., 2002,<br>Xiong and Yang, 2003,<br>Zhang et al., 2012b,<br>Jalmi and Sinha, 2016,<br>Song and Goodman, 2002<br>Xie et al., 2014 |
| MAPK<br>(B group) | ***OsMPK14* (*OsMPK3, OsMAPK3, OsMAP3, OsMAPKK33*)** | GQ265780 | AtMPK4, OsMPK6, AtMPK16 | low temperature$^{U}$, high salinity $^{U}$, drought $^{D}$, light in roots $^{U}$,<br>light in shoots $^{D}$ | OsMKK3 | Liang et al., 2010,<br>Lee et al., 2011,<br>Wankhede et al. 2013 |
| MAPK<br>(D group) | ***OsMPK17-1* (*OsBWMK1, OsMPK12*)** | AF177392 | OsRMAPK2, AtMPK9 | JA$^{U}$,SA$^{U}$ | - | Agrawal et al., 2003b |
| MAPK<br>(D group) | ***OsMPK20-4* (*OsWJUMK1, OsMPK8, OsMPKG1*)** | AJ512643 | OsMAPK44, AtMPK20, AtMPK18 | $H_2O_2^{U}$, heavy metals $^{U}$, cold stress $^{U}$ | OsMKK6, OsMPK3 | Agrawal et al., 2003a,<br>Sheikh et al., 2012 |
| MAPK<br>(D group) | ***OsMPK7 (OsMAPK44)*** | AY506572 | OsMPK20-4, AtMPK20 | NaCl$^{U}$, drought$^{U}$, $H_2O_2^{U}$, bacterial pathovar (*X. oryzae*)$^{U}$ | OsMKK3 | Jeong et al., 2006,<br>Zhang et al., 2014e,<br>Jalmi and Sinha, 2016 |
| MAPKKK<br>(Raf-like) | ***OsEDR1* (*OsMAPKKK1*)** | AY167575 | AtEDR1, HvEDR1 | wounding$^{U}$, JA$^{U}$, SA$^{U}$, ET$^{U}$, $H_2O_2^{U}$ | - | Kim et al., 2003 |
| MAPKKK<br>(Raf-like) | ***OsDSM1*(*OsMAPKKK6*)** | LOC_Os02g50970 | AtEDR1, AtCTR1 | NaCl$^{U}$, drought$^{U}$ | - | Ning et al., 2010 |
| ***Reaumuria soongorica*** | | | | | | |
| MAPK<br>(C group) | ***RsMPK2*** | *EU846599* | *PsMPK2,* NtNTF3, PsMPK2, PhMEK1 | $H_2O_2^{U}$, PEG$^{U}$ | - | Liu et al., 2010 |
| ***Solanum lycopersicum* (tomato)** | | | | | | |
| *MAPK* | ***SlMAPK3*** | - | - | $H_2O_2^{U}$ ,MeJA$^{U}$ , SA$^{U}$ | - | Li et al., 2017d |
| ***Solanum tuberosum* (potato)** | | | | | | |

| | | | | | | |
|---|---|---|---|---|---|---|
| MAPK (A group) | ***StMPK1*** | - (98% identical to an EST sequence ( AB062138) | *LeMPK1, SIPK,* AtMPK6, OsMPK6 | JA$^{U}$, wounding$^{U}$, pathogenic fungus (Fusarium Oxysporum f. Sp)$^{U}$ and heat stress$^{U}$ | - | Blanco et al., 2006 |
| ***Zea mays* (maize)** | | | | | | |
| MAPKK (A group) | ***ZmMKK1*** | *AB062138.1* | OsMKK1, AtMKK1, NtMEK1 | NaCl$^{U}$, ETH$^{U}$, PEG$^{U}$, $H_2O_2$ , 4°C $^{U}$, | ZmMEKK1 | Cai et al., 2014 |
| MAPKK (B group) | ***ZmMKK3*** | *JN972438* | OsMKK3, AtMKK3 | $H_2O_2^{U}$ , PEG$^{U}$ | - | Zhang et al., 2012d |
| MAPK (A group) | **ZmMPK6-2 (*ZmMPK5*)** | *AB016802* | AtMPK6, OsMPK1 | BR$^{U}$, SA$^{U}$, ETH$^{U}$, $H_2O_2^{U}$, $CdCl_2^{U}$, UV$^{U}$,light$^{U}$, hexavalent chromium (VI)$^{U}$, cold$^{U}$, NaCl$^{U}$ wounding stress$^{U}$ | - | Zhang et al. 2006, 2007, 2010; Lin et al., 2009; Ding et al. 2013;Sun et al., 2015; Ma et al., 2016 |
| MAPK (D group) | ***ZmMPK6*** | NM_001111768 | OsMPK15, OsMPK16-1, TaMPK2, GhMPK16, AtMPK16 | SA$^{U}$, $H_2O_2^{U}$, PEG$^{U}$, NaCl$^{U}$ | - | Sun et al., 2015 |
| MAPK (C group) | ***ZmMPK7*** | *EU616650* | OsMSRMK, AtMPK17 | $H_2O_2^{U}$ | - | Zong et al., 2009 |
| MAPK (C group) | ***ZmMPK8*** | - | OsMPK16, OsMPK17, AtMPK8, AtMPK15 | drought stress$^{U}$ | - | Sun et al., 2015 |
| **DOWN-REGULATED GENES**<br>***Brachypodium distachyon* (purple false brome)** | | | | | | |
| *MAPKK (A group)* | ***BdMKK6.2*** | *JX297555* | OsMPK6,AtMPK6, AtMPK10 | $H_2O_2^{U}$ , cold$^{U}$, heat$^{U}$ and PEG$^{D}$ | - | Chen et al., 2012; Sun et al., 2016 |
| ***Cucumis sativus L.* (cucumber)** | | | | | | |
| MAPK (A group) | ***CsNMAPK*** | DQ812086 | GmMAPK1, PsMAPK3, MsMAPK, StWIPK, LeMAPK, CaMAPK1 | $H_2O_2^{U}$ ,SA$^{U}$ and cold stress$^{D}$ | - | Xu et al., 2008b |
| ***Malus domestica (apple)*** | | | | | | |
| MAPK (A group) | ***MdMKK1*** | GU139793 | PcMKK2, NtSIPKK, LeMEK1, AtMKK2 | - | MdMPK1 | Wang et al., 2010a |
| ***Manihot esculenta* (cassava)** | | | | | | |
| MAPK (C group) | ***MeMAPK4*** | - | CsMPK1, SIMAPK9 | *osmotic*$^{D}$, *$H_2O_2^{D}$, NaCl*$^{U}$ , cold stress$^{D}$ | - | Yan et al., 2016 |
| MAPK (D group) | ***MeMAPK19*** | **-** | CsMPK9-2 | *osmotic*$^{D}$, *$H_2O_2^{D}$,* cold$^{D}$ and *NaCl*$^{U}$ | - | Yan et al., 2016 |
| ***Musa acuminata L.* (banana)** | | | | | | |

| | | | | | | |
|---|---|---|---|---|---|---|
| MAPK (D group) | ***MaMAPK1*** | *KF582545* | AtMPK20 | ETH$^{U}$, MeJA$^{U}$, SA$^{U}$, pathogenic fungus( Fusarium Oxysporum f. Sp)$^{U}$ | - | Wang et al., 2015b |
| ***Rheum australe D.*** | | | | | | |
| MAPK (A group) | ***RaMPK1*** | *DQ078119* | GmMPK2, PcMPK6, NTF4-2, StMPK, MsMMK1, LeMPK2, NtSIPK, NbSIPK, AtMPK, OsMPK6 | SA$^{D}$**,** JA$^{U}$ | - | Ghawana et al., 2010 |
| ***Zea mays* (maize)** | | | | | | |
| MAPKK (group C) | ***ZmMKK4*** | *EU954741* | *MsSIMKK, AtMKK5, AtMKK4, NtMEK2* | $H_2O_2$$^{U}$, cold(4°C)$^{U}$ PEG, NaCl$^{U}$ | - | Kong et al., 2011 |
| MAPK (group B) | ***ZmSIMK1*** | AY433815 | AtMPK4, AtMPK11 | NaCl$^{U}$, SA$^{U}$, $H_2O_2$, drought stress$^{U}$ | - | Gu et al., 2010 |

*Brassica Database (BRAD) http://brassicadb.org/brad
